# Electroacupuncture facilitates the integration of a grafted *TrkC*‐modified mesenchymal stem cell‐derived neural network into transected spinal cord in rats via increasing neurotrophin‐3

**DOI:** 10.1111/cns.13638

**Published:** 2021-03-24

**Authors:** Yang Yang, Hao‐Yu Xu, Qing‐Wen Deng, Guo‐Hui Wu, Xiang Zeng, Hui Jin, Lai‐Jian Wang, Bi‐Qin Lai, Ge Li, Yuan‐Huan Ma, Bin Jiang, Jing‐Wen Ruan, Ya‐Qiong Wang, Ying Ding, Yuan‐Shan Zeng

**Affiliations:** ^1^ Key Laboratory for Stem Cells and Tissue Engineering Ministry of Education Sun Yat‐sen University Guangzhou China; ^2^ Department of Histology and Embryology Zhongshan School of Medicine Sun Yat‐sen University Guangzhou China; ^3^ Guangdong Provincial Key Laboratory of Brain Function and Disease Zhongshan School of Medicine Sun Yat‐sen University Guangzhou China; ^4^ Department of Acupuncture The 1st Affiliated Hospital Sun Yat‐sen University Guangzhou China; ^5^ Department of Electron Microscope Zhongshan School of Medicine Sun Yat‐sen University Guangzhou China; ^6^ Co‐innovation Center of Neuroregeneration Nantong University Nantong China; ^7^ Institute of Spinal Cord Injury Sun Yat‐sen University Guangzhou China

**Keywords:** electroacupuncture, implantation, mesenchymal stem cells, neural tracing, neurotrophin‐3, spinal cord injury, tissue engineering neural network, tyrosine kinase C

## Abstract

**Aims:**

This study was aimed to investigate whether electroacupuncture (EA) would increase the secretion of neurotrophin‐3 (NT‐3) from injured spinal cord tissue, and, if so, whether the increased NT‐3 would promote the survival, differentiation, and migration of grafted *tyrosine kinase C* (*TrkC*)‐modified mesenchymal stem cell (MSC)‐derived neural network cells. We next sought to determine if the latter would integrate with the host spinal cord neural circuit to improve the neurological function of injured spinal cord.

**Methods:**

After *NT‐3*‐modified Schwann cells (SCs) and *TrkC*‐modified MSCs were co‐cultured in a gelatin sponge scaffold for 14 days, the MSCs differentiated into neuron‐like cells that formed a MSC‐derived neural network (MN) implant. On this basis, we combined the MN implantation with EA in a rat model of spinal cord injury (SCI) and performed immunohistochemical staining, neural tracing, electrophysiology, and behavioral testing after 8 weeks.

**Results:**

Electroacupuncture application enhanced the production of endogenous NT‐3 in damaged spinal cord tissues. The increase in local NT‐3 production promoted the survival, migration, and maintenance of the grafted MN, which expressed NT‐3 high‐affinity TrkC. The combination of MN implantation and EA application improved cortical motor‐evoked potential relay and facilitated the locomotor performance of the paralyzed hindlimb compared with those of controls. These results suggest that the MN was better integrated into the host spinal cord neural network after EA treatment compared with control treatment.

**Conclusions:**

Electroacupuncture as an adjuvant therapy for *TrkC*‐modified MSC‐derived MN, acted by increasing the local production of NT‐3, which accelerated neural network reconstruction and restoration of spinal cord function following SCI.

## Graphical abstract

A schematic diagram showing that MSC‐derived neural network is better integrated with the host spinal cord neural network after the EA treatment.

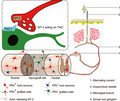

## 1 INTRODUCTION

Spinal cord injury (SCI) results in damage to spinal cord neurons, disrupting axonal tracts, and causing the dysfunction or loss of locomotor function, sensation, and autonomic functions below the injured spinal cord segments. To date, no effective therapy has been established for SCI. This is likely due to the multidimensional pathophysiological changes and the obstructive microenvironment that develop in the injury region.^1^ A combinatory treatment may result in a better therapeutic response relative to monotherapy by addressing multiple pathological aspects or mechanisms associated with SCI, such as inflammatory reactions, glial scar formation, and the insufficient supply of neural growth factors.^2^, ^3^ For functional recovery after SCI, a potential therapeutic strategy that has been proposed is the fabrication of a neuronal relay between the injured axonal tract projection fibers and the denervated neurons, which represents a necessary primary step for repairing damaged neural connectivity.

To achieve this goal, implantation of exogenous stem cell‐derived neurons into the injured spinal cord tissue has been the focus of many studies. This is because it is highly unlikely that the lost neurons can be replaced by the host neural stem cells, which tend to differentiate into neuroglial cells at the injury sites.^4^, ^5^ The direct transplantation of exogenous stem‐cell‐derived neurons or neuronal progenitor cells into the injured spinal cord may help compensate for and replace lost host neurons. Mesenchymal stem cells (MSCs), which are easy to obtain, ethically uncomplicated, and relatively safe, have emerged as a promising candidate for SCI treatment, with the potential to differentiate into neuron‐like cells. Although this approach remains controversial, experimental evidence has suggested that MSCs, after suitable modifications, can display phenotypic and functional neuronal properties.^6^, ^7^ We have previously reported that an MSC‐derived neural network (MN) implantation strategy was able to facilitate the recovery of limb motor function in a rat model following thorough spinal cord transection.^8^ Although the integration of some MSC‐derived neuron‐like cells was observed following the application of the MN to the host spinal cord tissue, the reconstruction of disrupted neural circuits was limited. Critical limitations that must be addressed to improve this experimental paradigm included poor survival and a low neuron‐like differentiation rate of the transplanted, neurotrophin‐3 (NT‐3) high‐affinity receptor tyrosine kinase C (TrkC)‐overexpressing MSCs within the host spinal cord tissue. The low level of NT‐3 within the local spinal cord environment may have impaired the message relay capabilities of the implanted MN scaffold. The loss of neurons is likely responsible for the permanent functional deficits associated with SCI; therefore, increasing the survival rate and differentiation of implanted MSC‐derived neuron‐like cells to restore and improve the disrupted neural circuit represents a logical approach to SCI treatment.

Electroacupuncture (EA), which is a traditional Chinese medicinal therapy, has been widely used and applied during clinical practice and in animal models for the treatment of SCI.^9^, ^10^, ^11^, ^12^, ^13^, ^14^ The application of EA has been demonstrated to have neural protective and anti‐inflammatory effects, which would improve the suitability of the damaged areas for axonal regeneration after SCI. In addition, because the NT‐3 levels in the injured spinal cord may represent a supportive factor during SCI repair, the potential that EA might enhance NT‐3 levels may support EA as a potential therapeutic strategy for SCI. The differentiation rate of donor NSCs and the induction of MSCs into neuron‐like cells at the site of SCI were reported to improve significantly following the application of EA.^11^, ^15^, ^16^ However, implanted prototype MSCs exhibited a low rate of development into neuron‐like cells.

These findings suggested that the combination of tissue engineering, such as neural network transplantation, together with EA therapy may represent an optimal approach for achieving increased survival and neuron‐like differentiation among implanted MSCs, which we explored in this study. We report here that the application of EA can increase the NT‐3 contents in transected spinal cord tissue, especially in the host tissue adjacent to the injury/graft site. More importantly, EA promoted the survival, differentiation, and migration of donor MSC‐derived neuron‐like cells, which is essential for the improved formation of potential neuronal relays at the injury/graft site of the spinal cord.

## 2 EXPERIMENTAL PROCEDURES

### 2.1 Animal care

Eighty‐four Sprague‐Dawley (SD) rats (Adult, female, 220–250 g) were used for all animal experiments in this study. All experimental protocols and animal handling procedures were approved by the Animal Care and Use Committee of Sun Yat‐sen University and were consistent with the National Institutes of Health Guide for the Care and Use of Laboratory Animals and were conducted in accordance with the *In Vivo* Experiments (ARRIVE) guidelines for Animal Research.^17^

### 2.2 Isolation and culture of MSCs and SCs

Mesenchymal stem cells were isolated, as previously described, from green fluorescent protein (GFP) transgenic SD rats (Osaka University, Osaka, Japan), which ubiquitously express GFP in all tissues.^18^ Briefly, one‐week‐old rats (*n* = 10) were sacrificed, their femurs were removed, and flushed of bone marrow, which was cultured in low‐glucose Dulbecco's modified Eagle medium (L‐DMEM, Gibco), supplemented with 10% fetal bovine serum (FBS, TBD Co, Tianjin, China) and 4 mM L‐glutamine (Invitrogen, USA), in a 5% CO_2_ incubator at 37°C. When adherent cells reached 80% confluence, they were passaged (1:3) into different culture flasks. MSCs from passages 3 to 5 were used for all experiments in this study.

To isolate Schwann cells (SCs), five‐day‐old neonatal SD rats (not GFP transgenic rats, *n* = 30) were decapitated and sterilized. The sciatic nerves and brachial plexus were dissected, and the adherent connective tissue and epineurium were removed under a dissecting microscope. The nerves were cut into small pieces (< 2 mm) and digested with 0.16% collagenase (Sigma‐Aldrich, St. Louis, MO) at 37°C for 15 min. The dissociated tissue was placed on culture dishes coated with poly‐L‐lysine and containing DMEM/F12 medium containing 10% FBS for 30 min at 37°C in a 5% CO_2_ humidified atmosphere. After 30 min, an additional 2 ml of medium was added to each culture dish. The medium was changed every 2 days. After 5–7 days, when the cells reached 80% confluency, they were subcultured and purified using differential velocity adherent methods. Based on our recent study, 95–96% of the cells were estimated to be SCs.^19^

### 2.3 MSC and SC transfection and seeding on a 3D gelatin sponge scaffold

Briefly, *in vitro*, the MSCs and SCs were transduced with recombinant adenoviruses (Advs) containing the *TrkC* gene (Adv‐TrkC) and the *NT*‐*3* gene (Adv‐NT‐3), respectively, to induce the overexpression of TrkC and the oversecretion of NT‐3. After 3 h, Adv‐TrkC, administered at a multiplicity of infection (MOI) =300, yielded 81.32% transduced MSCs, whereas Adv‐NT‐3 administered at an MOI =100 yielded 78.19% transduced SCs, with excellent viability. When higher viral titers were used, the cells tended to show pathological changes. The medium was replaced with DMEM/F12 supplemented with 10% FBS, and the cells were incubated for 24 h at 37°C.

A three‐dimensional (3D) gelatin sponge scaffold with a 3‐mm diameter and a 2‐mm length was prepared, as previously described.^8^ Scaffolds were seeded with 1 × 10^5^ total cells (equal numbers of MSCs and SCs) to generate MNs and were cultured in 10 µl culture medium and incubated at 37°C for 14 days; the culture medium was changed every 2 days.

### 2.4 Spinal cord injury and transplantation

Three days before surgery, the rats received a subcutaneous cyclosporin A injection in the belly (1 mg/100 g per rat). They were anesthetized with 1% pentobarbital sodium (40 mg/kg, i.p.). A laminectomy was performed to expose the T9 and T10 spinal cord segments, and the dura was slit vertically with a pair of micro‐forceps and micro‐scissors. A pair of angled micro‐scissors was used to fully transect the spinal cord, and a 2‐mm segment of the spinal cord was removed at the T10 spinal cord level. Then, either the generated MNs (the MN group) or gelatin sponge scaffolds containing no cells (the GS group) were used to fill the spinal cord gap.^8^ After the surgical incisions were sutured, the rats received extensive postoperative care, including the intramuscular injection of penicillin (50,000 U/kg/day) for 3 days. The manual emiction was performed on the experimental rats twice daily until automatic micturition was re‐established. Cyclosporin A was administered once daily for 8 weeks.

### 2.5 Electroacupuncture

Five days post‐surgery, the rats in the GS+EA and MN+EA groups were subjected to EA treatment every other day for 8 weeks. EA stimulation was applied at two pairs of Governing Vessel (GV) acupoints: 1.) GV9 (Zhiyang) and GV6 (Jizhong); and 2.) GV2 (Yaoshu) and GV1 (Changqiang) (Figure S1). The locations of the GV acupoints were determined as described previously.^20^ GV1 is located at the midpoint between the tip of the coccyx and the anus in the prone position. GV2 is located on the posterior midline, in the depression below the spinous process of the fourth sacrum. GV6 is located on the posterior midline, in the depression below the spinous process of the eleventh thoracic vertebra in the prone position. GV9 is located on the posterior midline, in the depression below the spinous process of the seventh thoracic vertebra in the prone position. GV6 and GV9 are located in the depressions below the rostral and caudal spinous processes of the transected spinal cord, respectively. EA applied to both GV6 and GV9 can be used directly to treat the injured spinal cord. EA applied to both the GV1 and GV2 can improve bowel and bladder emptying and reduce the paralysis of the lower or hindlimbs. The rats were positioned using a specially designed restrainer, without anesthesia, which maintained them in a recumbent position during the EA treatment. Stainless steel acupuncture needles (0.30 mm in diameter, 50 mm in length; Jiangsu Medical Instruments Inc., China) were inserted at a depth of 5 mm into the GV acupoints.^16^ The two pairs of needles were connected to the output terminals of an EA apparatus (model number G6805‐2A, Shanghai Medical Electronic Apparatus Company, China). EA was applied using alternating strings of dense sparse waves at alternating frequencies (60 Hz for 1.05 s and 2 Hz for 2.85 s, pulse width of 0.5 ms). The positive and negative electrodes used to connect the two pairs of needles were alternated with each treatment. During the EA process, the current intensity was tested in the animal's body between the acupoint pair across the graft location, which indicated a current intensity of approximately 5 µA.

### 2.6 Assessment of locomotor function

After surgery, the rat hindlimb function was assessed weekly, using the Basso, Beattie, and Bresnahan (BBB) open‐field locomotor test^21^ to evaluate voluntary movement and body weight support and a modified inclined‐grid climbing test to qualitatively assess the accuracy of foot placement and coordination, which differentiates local reflex activity from voluntary movement. Two independent investigators blinded to the experimental treatments determined the BBB scores.

### 2.7 Electrophysiology

Evoked potentials (EPs) were measured and recorded (*n* = 5/group), as previously described, to evaluate motor axonal conduction.^22^ Under general anesthesia, the sensorimotor cortex (SMC) and sciatic nerve of rats were exposed. Stimulating electrodes (NeuroExam M‐800 Data Acquisition Analysis System, MEDCOM, Zhuhai, China) were placed in the SMC (located 2 mm lateral to the midline and 2 mm caudal to Bregma), and the recording electrodes were connected to the sciatic nerve. The amplitude of the cortical motor‐EP (CMEP) was calibrated and then recorded.

### 2.8 CTB retrograde tracing

Two months after surgery, the rats were anesthetized with 1% pentobarbital sodium (40 mg/kg, i.p.), and their sciatic nerves were exposed under sterile conditions. Using a dissecting stereomicroscope (Leica Microsystems, Inc., Wetzlar, Germany), the needle tip of a 30 g needle Hamilton syringe (Hamilton Co., Reno, USA) was inserted 10 mm into the sciatic nerve along its longitudinal axis and then withdrawn 2 mm to create a potential pool for injection. Then, 2 µl of 2% cholera toxin B (CTB) conjugated to Alexa Fluor 555 (CTB‐555, Life, USA) was slowly injected, as previously described.^23^ The sciatic nerve proximal to the injection site was lightly crushed with a pair of blunt forceps to facilitate CTB‐555 uptake by the nerve fibers. The injection site was thoroughly rinsed using a sterile saline‐soaked cotton‐tipped stick, and the wound was sutured. The rats were sacrificed 1 week after tracer injection.

### 2.9 Pseudorabies virus (PRV‐CMV‐mRFP) retrograde tracing

The same injection procedure described for CTB retrograde tracing was used to slowly inject 1 µl of pseudorabies virus (PRV, 2.5 × 10^9^ PFU/ml; Brainvta, China), after which the needle was maintained in place for 5 min. The sciatic nerve was lightly crushed with a pair of blunt forceps proximal to the injection site to maximize PRV contacts with the nerve fibers. The injection site was thoroughly rinsed with a sterile saline‐soaked cotton‐tipped stick, and the wound was sutured. Twenty rats (*n* = 5/group) were sacrificed 6 days after injection.

### 2.10 Tissue processing

Eight weeks after spinal cord transection, 20 rats (*n* = 5/group) were anesthetized and perfused transcardially with 200 ml of 0.1 M PBS. A 1‐cm long segment of the spinal cord, which included the injury/graft site, was mechanically homogenized in 0.1 M PBS. Homogenates were centrifuged for 10 min at 18800 g at 4°C and used for Western blot analysis. The relative expression of target protein was indicated by the target protein/GAPDH (loading control) intensity ratio as reported in a previous study.^24^

The rats were sacrificed at the end of 2 or 8 weeks following scaffold transplantation. All rats were deeply anesthetized with 1% pentobarbital sodium (50 mg/kg, i.p.) and transcardially perfused with normal saline containing 0.002% NaNO_2_ and 0.002% heparin, followed by a fixative containing 4% paraformaldehyde (PFA) in 0.1 M PBS (pH 7.4). The spinal cord was dissected and post‐fixed for 24 h in the same fixative, followed by 30% phosphate‐buffered sucrose at 4°C for 48 h. Samples were frozen and embedded in OCT compound. The T8–T12 successive segments of the spinal cord were cut into longitudinal 25‐µm‐thick sections.

### 2.11 Immunofluorescence staining

Specific proteins in the obtained spinal cord tissue sections were detected by immunofluorescence staining (IFS). Sections were immersed in 0.01 M phosphate‐buffered saline (PBS) three times for 5 min and then blocked with 10% goat serum for 30 min at 37°C. The sections were incubated with primary antibodies in 0.01 M PBS containing 0.3% Triton X‐100 at 4°C for 12 h. The tissue sections were washed with PBS three times for 5 min and incubated with secondary antibodies for 1 h at 37°C. The slides were observed, and images were captured using a fluorescence microscope (Leica) or confocal microscope (Carl Zeiss). A summary of the antibodies used can be found in Table S1.

### 2.12 *In situ* hybridization

Twenty‐five rats (*n* = 5/group) were transcardially perfused 2 weeks after SCI. The spinal cord was dissected from the T8 to T12 successive segments, post‐fixed in 4% paraformaldehyde for 2–4 h and saturated in 30% sucrose overnight at 4°C; 1% diethylpyrocarbonate was added to the above solution to prevent mRNA degradation. The tissue samples were cryosectioned into 20‐μm‐thick sections and stored at −20°C. The following procedures were performed according to the manufacturer's instructions (NT‐3 mRNA *in situ* hybridization kit, Boster, Wuhan, China). The sections were thawed, washed three times with *in situ* hybridization PBS, and incubated in blocking buffer (without probes) for 2–3 h at 42°C to reduce nonspecific hybridization. Sections were incubated with 20 µl hybridization solution containing a digoxin‐labeled NT‐3 mRNA oligonucleotide probe (No. MK1641‐r, Boster, Wuhan, China): 5’‐TCAAA GGAGT TTGCC AGAAG ACTCG CTCAA‐3’. The sections were rinsed with 2× SSC (NaCl 17.6 g, C_6_H_5_O_7_Na_3_ 8.8 g in 1000 ml ddH_2_O) for 15 min, 0.5× SSC for 15 min, and 0.2× SSC for 15 min. The sections were incubated with biotin for 60 min at 37°C, followed by incubation with streptavidin‐biotin complex (SABC) conjugated with Alexa Fluor 555 (SABC‐Alex555) for 1 h. After brief staining with Hoechst33342, the sections were covered with coverslips and examined under a fluorescence microscope.

### 2.13 Immunoelectron microscopy

For immunoelectron microscopy (IEM) analysis, two rats each in the MN+EA and MN groups were transcardially perfused with 0.1 mol/L sodium phosphate buffer containing 187.5 units/100 ml heparin, followed by perfusion with 4% PFA containing 0.1% glutaraldehyde and 15% saturated picric acid. The spinal cord segment containing the injury/graft site was post‐fixed overnight at 4°C in fresh 4% PFA and subsequently cut into 50‐µm‐thick sagittal sections on a vibratome. To improve the penetration of antibodies, vibratome sections were transferred into a cryoprotectant solution containing 25% sucrose and 10% glycerol in 0.1 M PBS overnight at 4°C, followed by three rapid freeze–thaw cycles in liquid nitrogen. After washing with PBS, the sections were treated for 1 h with 20% goat serum (Tris buffer, pH 7.4) to block nonspecific binding. The sections were incubated with primary antibodies (anti‐GFP, anti‐5‐hydroxytryptamine [5‐HT] or anti‐choline acetyltransferase [ChAT]) in 2% normal goat serum solution at 4°C for 24 h, followed by incubation with secondary antibody (1:300, 1.4 nm nano‐gold‐conjugated anti‐rabbit IgG, NanoProbes, USA) overnight at 4°C. Sections were post‐fixed in 1% glutaraldehyde for 10 min and enhanced with Gold Enhance TM EM Plus Kit (NanoProbes). When double‐labeling IEM was performed, the tissue slices were treated with 0.3% H_2_O_2_ to scavenge endogenous peroxidase before adding the blocking serum. The horseradish peroxidase (HRP)‐conjugated secondary antibodies (Vectastain ABC‐HRP Kits, Vector Laboratories, Burlingame, CA, USA) were visualized using 3, 3′‐diaminobenzidine (DAB, Vector Laboratories). The sections were osmicated with 2% OsO_4_, dehydrated in a graded ethanol series, and embedded in Epon812 for ultrathin sectioning. After staining with uranyl acetate, the sections were examined under a transmission electron microscope (Philips CM 10).

### 2.14 Morphological quantification

To quantify immunopositive cells *in vivo*, 10 separate sections from each animal were selected (*n* = 5/group). After IFS with respective antibodies, five areas (four corners and one center) were examined at 200× magnification. The percentage of immunopositive cells was obtained by dividing the total number of immunopositive, GFP, and Hoechst33342 triple‐positive cells by the total number of GFP and Hoechst33342 double‐positive cells.^8^

To observe the area the injury/graft site of the spinal cord, one in every nine longitudinal sections from each animal (a total of 10 sections per rat, *n* = 5/group) was stained with anti‐GFP antibodies and imaged at 100× magnification; the areas emitting detectable GFP‐positive signal at this magnification were outlined and measured using ImageJ software.

The quantitative analysis of 5‐HT‐positive axons was performed as described previously.^25^, ^26^ A calibrated reticle eyepiece was used to delineate regions 300 µm rostral to the injury site, at the injury site, and 300 µm caudal to the injury site (see reference^25^ and Additional file 1: PDF1 for a sketch of the injured spinal cord that was used to clarify the quantification location). The 5‐HT‐positive axon profiles were quantified in all regions, at 200× magnification. The spinal cord was cut in longitudinal sections, and every fifth section was mounted on a gelatin‐coated slide. Ten sections per rat were analyzed, and the total number of 5‐HT‐positive nerve fibers in all regions of each experimental group was averaged. The nerve fibers exceeding 25 µm were counted as 5‐HT‐positive axons.

To analyze the PRV‐labeled cells in the T9, T10, and T11 segments of the spinal cord, one in every nine longitudinal sections from each animal (a total of 10 sections per rat, *n* = 5/group) was stained with anti‐microtubule‐associated protein 2 (Map2) antibody and imaged at 100× magnification. Positive cells were counted using ImageJ software.

For the quantification of NT‐3 mRNA or NT‐3 protein *in vivo*, positive cells were quantified using ImageJ, as described previously.^27^ Setting the epicenter of the SCI site as the origin, the injury site, and adjacent areas were separated into eight regions. NT‐3 mRNA positive cells were converted into an area of interest (AOI), and the pixel area of each AOI was automatically calculated by ImageJ. The staining density for each region, representing the total pixels in each AOI, was then calculated.

### 2.15 Transwell migration assay

A chemotaxis assay examining the effect of NT‐3 on TrkC‐MSCs and MSCs was performed in 24‐well plates containing 8‐μm porosity inserts (Corning, NY, USA). The cells were washed twice with serum‐free DMEM and suspended at 5 × 10^5^/ml in DMEM containing 0.5% BSA. Cells (3.0 × 10^4^) in 100 µl were loaded onto the top well. Serum‐free DMEM containing 0, 20, 40, 60, or 80 ng/ml NT‐3 was added to the bottom chamber at a total volume of 0.6 ml. For the inhibition experiment, TrkC‐MSCs and MSCs were pre‐treated with 10 nM of the TrkC inhibitor K252a (Millipore, USA; K252a is an efficient serine/threonine‐protein kinase inhibitor). After 4 h incubation, the non‐migrating cells were completely removed from the top surface of the filters, and the migrating cells adhering to the undersurface of the filters were stained with Hoechst33342 and quantified with Image J.

### 2.16 Statistical analysis

All statistical analyses were performed using the statistical software SPSS version 20.0. Data are reported as the mean ±standard deviations (SD). The data were analyzed using one‐way analysis of variance (ANOVA). The Shapiro‐Wilk's test and Levene's test were conducted to confirm normality of the data. If equal variances were found, the least significant difference test was applied; otherwise, the data with heterogeneity of variance were analyzed using nonparametric test (Kruskal‐Wallis test). When two groups were compared, the unpaired Student's *t*‐test was used. *p* < 0.05 was considered significant, and *p* < 0.01 was considered highly significant.

## 3 RESULTS

### 3.1 TrkC‐MSCs exhibit increased survival rates and migration following EA

**FIGURE 1:**
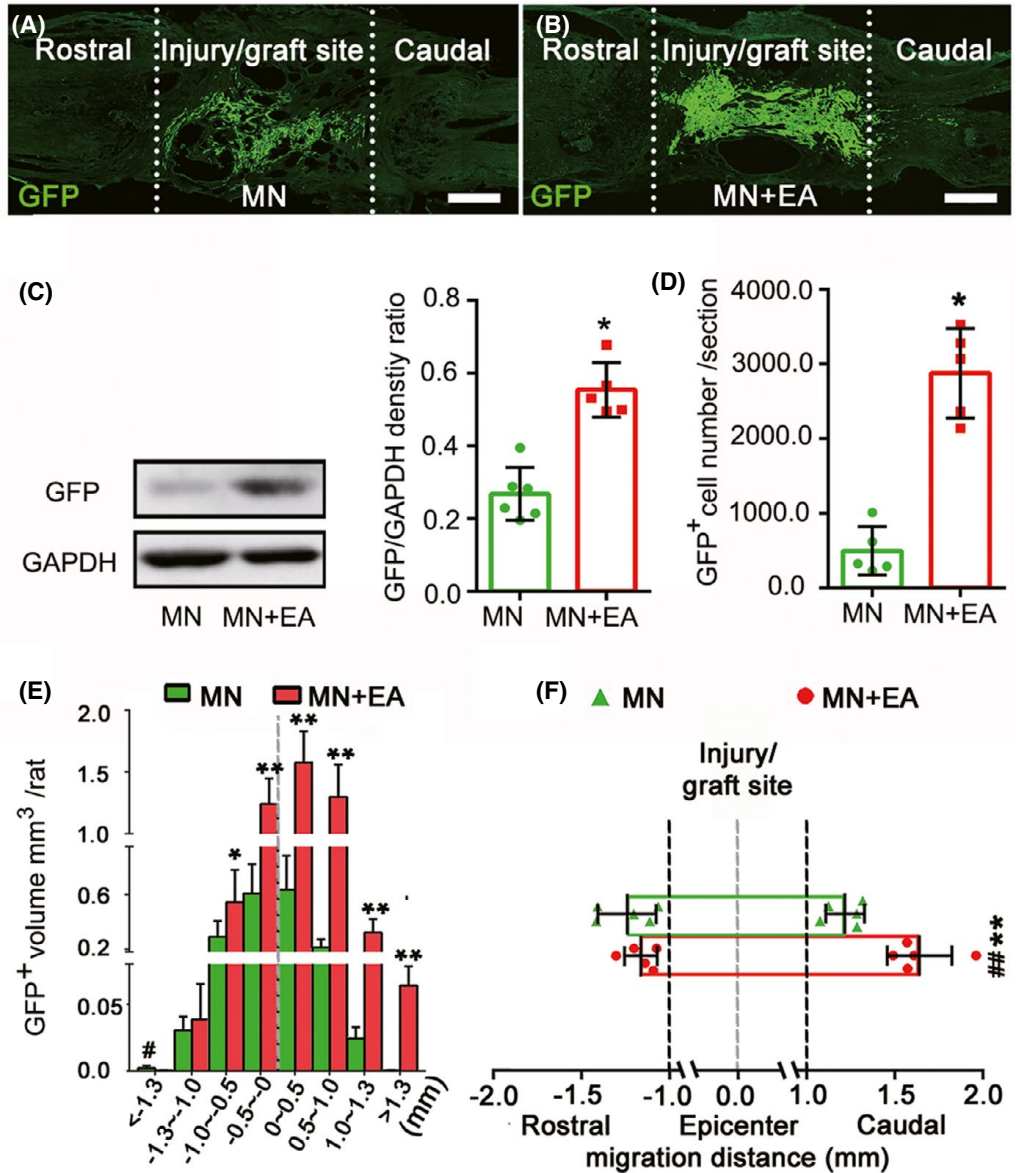
Electroacupuncture (EA) promoted the survival and migration of mesenchymal stem cell (MSC)‐derived cells *in vivo*. (A, B) Representative immunofluorescence images of injured spinal cord sections from the MSC‐derived neural network (MN, A) and MN+EA groups (B) at 8‐week post‐injury (wpi). Scale bars = 500 µm. Green fluorescent protein‐positive (GFP^+^) cells (green) are MSC‐derived cells from the grafted MN. (C) Western blot analysis of GFP expression in the injury/graft site in the MN+EA and MN groups at 2 wpi (*n* = 5/group). (D, E) Bar charts showing the number (D) and volume (E) of grafted GFP^+^ cells. Values represent the mean ± SD (*n* = 5/group, Student's *t*‐test, *p < 0.05). (F) Graphical representation showing the average maximum migration distance of GFP^+^ cells from the epicenter of grafted MN to the rostral and caudal sides in each group. The quantification of migration distance was performed using ImageJ. Values represent the mean ± SD (*n* = 5/group. *p < 0.05, **p < 0.01, and ^##^ *p* < 0.01, determined by unpaired Student's *t*‐test). GAPDH, glyceraldehyde 3‐phosphate dehydrogenase

To assess the survival of transplanted MSC‐derived cells **(**GFP‐positive cells) at 8‐week post‐injury (wpi), the area occupied by GFP‐positive cells was measured at low magnification. In the MN group, only a few GFP‐positive cells were distributed in the injury/graft site of the spinal cord 8 weeks after MN transplantation (Figure 1A). However, in the MN+EA group, a larger number and volume of GFP‐positive cells were observed in the injury/graft site, and some cells migrated into the area caudal to the grafted site (Figure 1B). Additionally, western blot analysis showed that GFP expression in the MN+EA group was significantly increased compared with that in the MN group at 2 wpi (Figure 1C).

The numbers, volume, and distribution differences in MSC‐derived cells in the injury site were compared between the MN and MN+EA groups. Sections were arranged by position, relative to the injury midline, which was defined as the most severely damaged section with the largest scar.^28^ The number and volume of GFP‐positive grafts in the MN+EA group were larger than those in the MN group (Figure 1D,E). In the MN group, GFP‐positive MSCs were confined primarily to the injury/graft site of the spinal cord and rarely migrated beyond the injury/graft site (Figure 1E). In contrast, many GFP‐positive MSCs were distributed in the region caudal to the damage epicenter in the MN+EA group. The proportions of GFP‐positive MSCs in each section were significantly increased in the MN+EA group compared with those in the MN group (Figure 1E).

The longest migration distance for GFP‐positive MSC‐derived cells relative to the injury epicenter was analyzed to determine the migration range. The GFP‐positive MSC cohort exhibited a migration range of 1242.20 ± 164.20 µm rostral and 1290.20 ± 104.90 µm caudal to the injury epicenter, with the largest total range of 2801.50 µm, in the MN group (Figure 1F). In the MN+EA group, the GFP‐positive MSC cohort showed a migration range of 1210.10 ± 96.60 µm rostral and 1640.10 ± 184.70 µm caudal to the injury epicenter, with the largest total range of 3131.50 µm (Figure 1F). On closer analysis, GFP‐positive MSCs migrated preferentially into the adjacent area caudal to the injury/graft site over the adjacent rostral area (*p* < 0.01) in the MN+EA group. The migration distance of GFP‐positive MSCs into the adjacent area caudal to the graft site was larger in the MN+EA group than that in the MN group (*p* < 0.01). In addition, no significant difference was observed between the rostral and caudal migration distances of GFP‐positive MSCs in the MN group (Figure 1F, *p* > 0.05).

### 3.2 Donor MSCs differentiate preferentially into neuron‐like cells after EA treatment

Eight weeks after implantation into the injured spinal cord, some donor MSCs expressed NSC (Nestin) and immature neuron markers (doublecortin [DCX] and β‐tubulin III, Figure S2A–L). Occasional donor MSCs in the MN group exhibited expression of the mature neuron marker neurofilament‐h (NF‐H, Figure S2M,N). Following EA treatment, more donor MSCs differentiated into neuron‐like cells expressing neuronal markers (NF‐H, Figure S2O,P) than in the MN group; a small number of MSCs maintained Nestin (Figure S2C,D) and DCX (Figure S2G,H) expression. Some MSCs in the MN+EA group expressed β‐tubulin III (Figure S2K,L).

The quantification of neuron markers showed that EA treatment resulted in significantly more NF‐H‐positive donor MSC cells (37.71 ± 4.13% *vs*. 10.01 ± 2.65%) and fewer cells expressing the immature neuron markers β‐tubulin III (61.27 ± 3.46% *vs*. 74.22 ± 5.84%) and DCX (19.72 ± 11.78% *vs*. 24.92 ± 5.98%) or the NSC marker Nestin (22.48 ± 2.01% *vs*. 31.20 ± 2.53%) compared with those in the MN group (Figure S2G–Q).

### 3.3 Formation of synapse‐like structures between MSC‐derived neuron‐like cells

**FIGURE 2:**
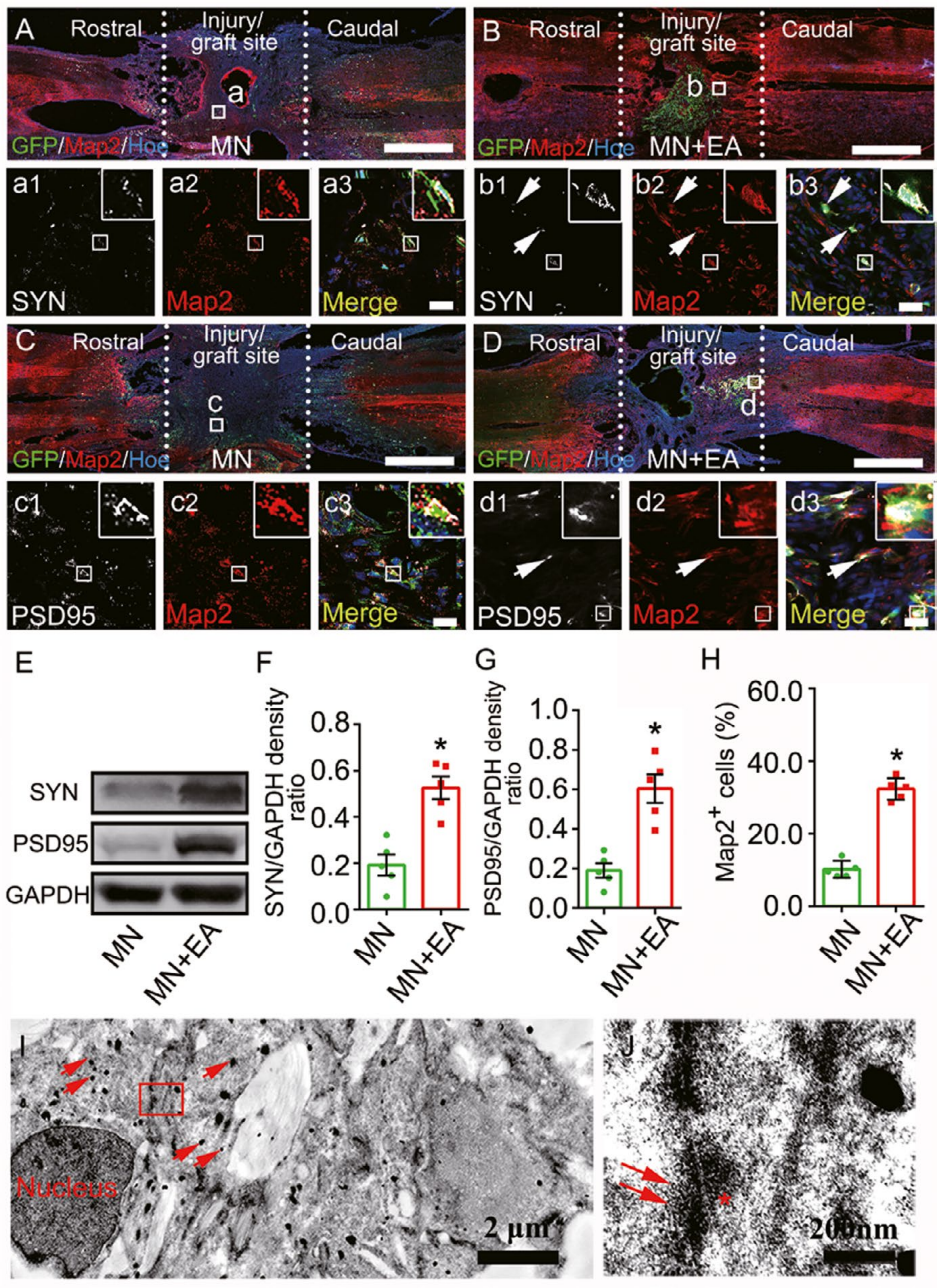
Mesenchymal stem cell (MSC)‐derived neuron‐like cells and synapse‐like structures in the graft site of the spinal cord at 8 wpi. (A–D) Representative images triple‐labeled with anti‐green fluorescent protein (GFP, green), microtubule‐associate protein (Map2, red; a2,b2,c2, and d2) and synapsin (SYN, white; a1 and b1) or postsynaptic density protein 95 (PSD95, white; c1 and d1) in the epicenter of the injury/graft site. The merged images show the GFP^+^ cells that colocalize with Map2/SYN (arrows, a3 and b3) or Map2/PSD95 (arrows, c3 and d3). The cell nuclei were counterstained with Hoechst33342 (Hoe). (E–G) Western blot analysis of SYN and PSD95 expression in the injury/graft site of the spinal cord. Values represent the mean ± SD (*n* = 5/group, *p < 0.05). (H) Bar chart showing the percentage of GFP^+^ cells that differentiated into Map2^+^ neuron‐like cells. Values represent the mean ± SEM (*n* = 5/group, *p < 0.05). (I, J) IEM revealed that a GFP^+^ cell, labeled by silver‐enhanced nanogold particles (arrows), formed a synapse‐like structure with another GFP^+^ cell. The structure was characterized by the presence of some synaptic vesicles (J, arrows) of varying diameters in the “presynaptic element,” electron dense material in the “postsynaptic element” (J, asterisk), and a “synaptic cleft” between the presynaptic and postsynaptic elements. Scale bars = 1 mm in (A–D); 20 µm in (a1)–(a3), (b1)–(b3), (c1)–(c3) and (d1)–(d3)

To assess whether EA could promote the *de novo* formation of synaptic contacts between the transplanted MSC‐derived neuron‐like cells, western blot, immunofluorescence staining, and immunoelectron microscopy assays were performed. The results showed that donor Map2‐positive neuron‐like cells displayed only moderate expression levels of synaptophysin (SYN) in the injury/graft site of the spinal cord in the MN group (Figures 2A, a1‐3); however, in the MN+EA group, many donor Map2‐positive neuron‐like cells emitted intense SYN‐positive immunofluorescence (Figures 2B, b1‐3). Similarly, compared with the MN group (Figures 2C, c1‐3), more donor Map2‐positive neuron‐like cells expressing postsynaptic density protein 95 (PSD95) were observed in the injury/graft site in the MN+EA group (Figures 2D, d1‐3). Western blot analysis showed that the expression levels of SYN and PSD95 proteins in the injury/graft site of the MN+EA group were significantly higher than those in the MN group (Figure 2E–G, *p < 0.05).

To detect the effects of EA on MSC differentiation *in vivo*, we quantified the percentage of cells expressing the mature neuron marker Map2. The results revealed that the percentage (34.30 ± 3.10%) of Map2^+^GFP^+^ cells in the MN+EA group was significantly higher than that (10.12 ± 2.40%) in the MN group (Figure 2H, *p < 0.05). Furthermore, MSC‐derived neuron‐like cells appeared to form synapse‐like structures with each other in the MN+EA group, as confirmed by IEM (Figure 2I and J). These results suggested that EA may promote the formation of synapse‐like structures between transplanted MSC‐derived neuron‐like cells *in vivo*.

At 8 weeks after EA treatment, 19.70 ± 3.67% of transplanted Map2‐positive cells expressed gamma‐aminobutyric acid (GABA, Figure S3A,B,G), 8.03 ± 3.09% of the cells expressed ChAT (Figure S3C,D,G), and 67.73 ± 5.78% of the cells expressed calmodulin‐dependent protein kinase II (CaMKII, Figure S3E,F,G). These findings indicated that the transplanted MSC‐derived neuron‐like cells possessed both excitatory and inhibitory characteristics.

### 3.4 MSC‐derived neuron‐like cells integrate into host neural circuits

**FIGURE 3:**
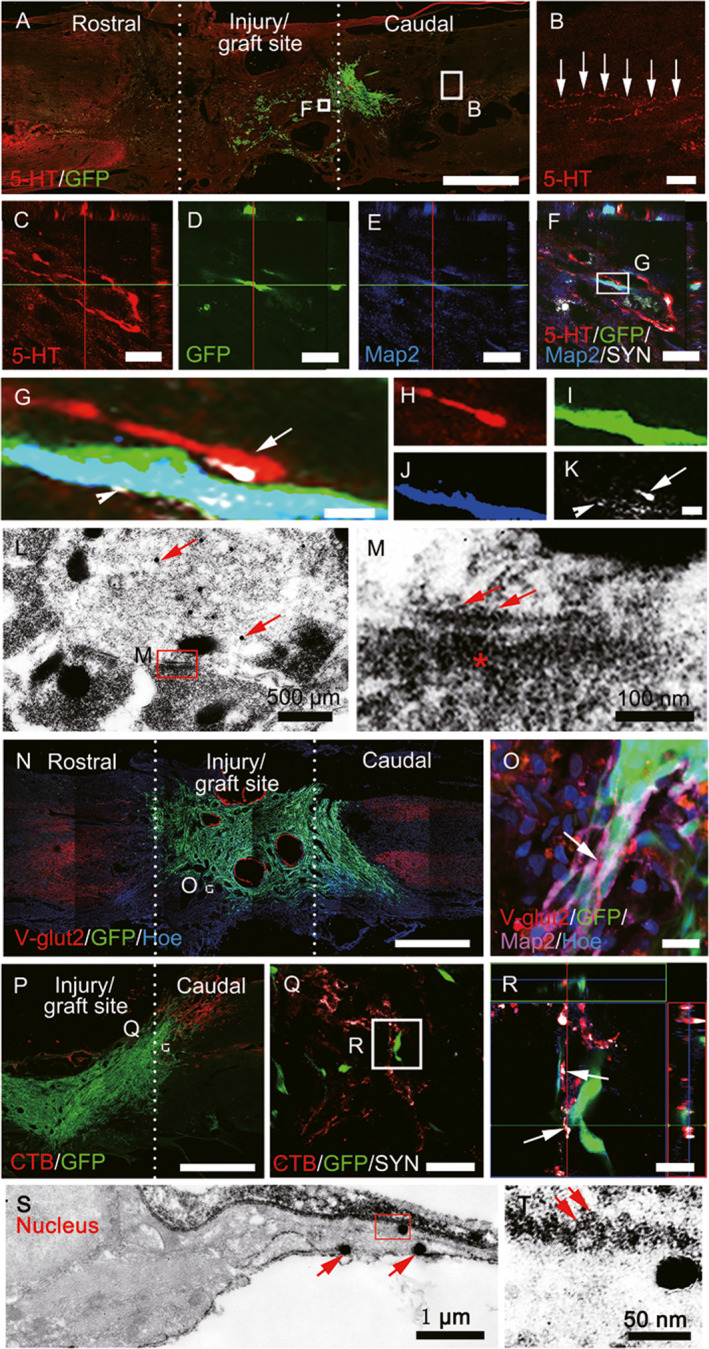
Mesenchyma stem cell (MSC)‐derived neuron‐like cells integrated into host neural circuits. (A, B) A few 5‐hydroxytryptamine (5‐HT)^+^ nerve fibers regenerated into the injury/graft site of the spinal cord. (C–K) Green fluorescent protein (GFP)/5‐HT/microtubule‐associated protein 2 (Map2)/synaptophysin (SYN) quadruple‐labeled immunostaining showing 5‐HT^+^ axon terminals in close apposition to GFP/Map2‐positive neuron‐like cells. (G–K) Representative magnified images of the boxed area in (F), quadruple‐labeled with anti‐5‐HT (red, H), GFP (green, I), Map2 (blue, J), and SYN (white, H and K), and their merged image (H). (L) Immunoelectron microscopy (IEM) revealed that 5‐HT^+^ axons stained with silver‐enhanced nanogold particles (arrows) formed synapse‐like structures with transplanted GFP‐positive cells (stained by 3, 3′‐diaminobenzidine [DAB]). The synapse‐like structures are characterized by the presence of some “synaptic vesicles” (arrows) of varying diameters in the cytoplasm of one of the processes in (M), electro‐dense material in the “postsynaptic element” (asterisk) in (M), and a narrow intercellular space between these elements. (N) and (O) Some vesicular glutamine transporter 2 (V‐Glut2)^+^ boutons (R, arrows) made contacts with GFP^+^/Map2^+^ neuron‐like cells in the injury/graft site of the spinal cord. (P–R) CTB^+^ ascending nerve fibers (Q) contacted GFP^+^ cells, featuring bouton‐like terminals (R). (S, T) IEM revealed that GFP^+^ cells stained with silver‐enhanced nanogold particles (S, arrows) could form synapse‐like structures with host axons (DAB^+^, arrows, T). Scale bars = 1 mm in (A), (N), and (P); 100 µm in (B); 50 µm in (Q); 20 µm in (C)–(F), (O), and (R); 1 µm in (G)–(K)

After SCI, 5‐HT positive axons in the graft site of the spinal cord were interrupted. In the MN+EA group, GFP/5‐HT/Map2/SYN quadruple‐labeled immunostaining showed that a 5‐HT‐positive axon terminal (button‐like) formed synaptic‐like contacts with a GFP/Map2‐positive neuron‐like cell (Figure 3A–K). By IEM, 5‐HT‐positive axons, stained with silver‐enhanced nanogold particles, appeared to form synapse‐like contacts with transplanted GFP‐positive cells (Figure 3L,M). The synapse‐like structure was characterized by the existence of some synaptic vesicles of varying diameters in the axonal terminal (Figure 3M). Vesicular glutamate transporter 2 (V‐glut2) is a descending excitatory nerve fiber marker, including in the rubrospinal and propriospinal tracts. V‐glut2‐positive axons also regenerated in the injury/graft site of the spinal cord and formed close contacts with GFP/Map2‐positive MSC‐derived neuron‐like cells in the MN+EA group (Figure 3N,O).

To ascertain whether the ascending regenerating axons could form contacts with transplanted MSC‐derived cells, CTB‐Alex555 was injected bilaterally into the sciatic nerve to retrogradely trace the ascending sensory nerve bundle. CTB/SYN‐positive axons and their boutons were in close association with Map2/GFP‐positive neuron‐like cells in the injury/graft site (Figure 3P–R). By IEM, in the area caudal to the graft site, a host axon (labeled by DAB) was found to form a synapse‐like structure with the grafted GFP‐positive neuron‐like cell (labeled by silver‐enhanced nanogold particles, Figure 3S). The synapse‐like structure contained some spherical vesicles in the presynaptic element formed by the host axon (Figure 3T). Compared with the MN group, the MN+EA group showed a larger number of CTB‐positive axons, many of which were distributed in the caudal lesion margin (Figure S4A–G). At 8 wpi in the MN+EA group, many MSC‐derived cells had migrated and made contacts with the CTB‐positive axons, which occurred more frequently than in the MN group (Figure S4C,F). Moreover, in the MN+EA group, the host nerve fibers had extended and were localized in the area caudal to the injury site (Figure S4H), where they often appeared to converge onto MSC‐derived neuron‐like cells. Very strikingly, many of these fibers converged on the processes of the MSC‐derived neuron‐like cells forming synaptic like contacts that were SYN‐positive (Figure S4H–M).

### 3.5 Pseudorabies virus tracing

**FIGURE 4:**
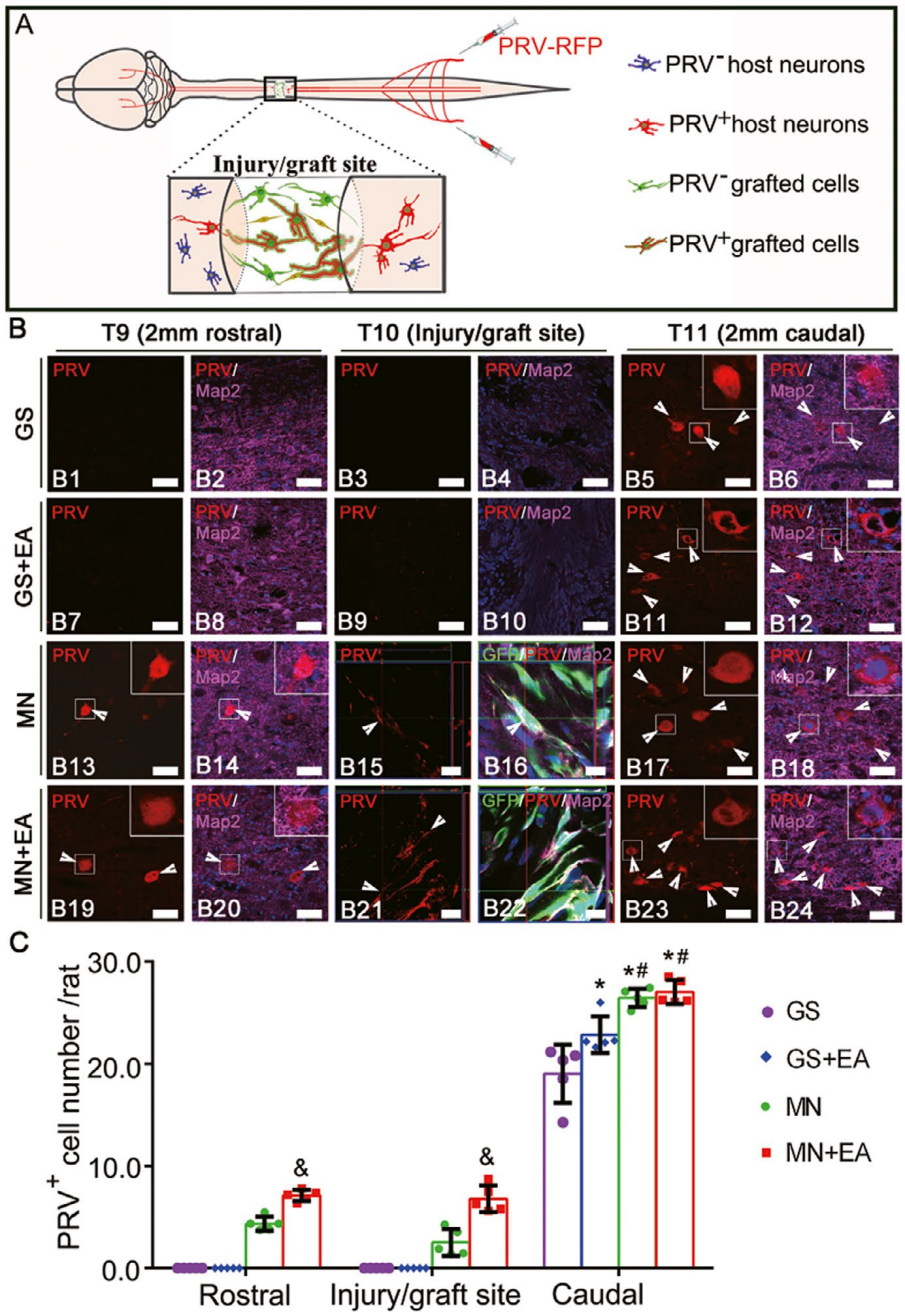
Pseudorabies virus (PRV) retrograde transsynaptic labeling confirmed the integration of transplanted mesenchymal stem cell (MSC)‐derived neuron‐like cells into the host spinal cord neuronal circuit. (A) A schematic diagram showing that PRV that was injected into the sciatic nerve was transported from the caudal area to the rostral area through the injury/graft site of the spinal cord. (B) Representative images showing the host neurons or MSC‐derived neuron‐like cells retrogradely labeled with PRV (red, arrowheads) in the rostral and caudal regions relative to the graft tissue of spinal cord in the GS group (B1–B6), GS+EA group (B7–B12), MN group (B13–B18) and MN+EA group (B19–B24). The cell nuclei were counterstained with Hoechst33342 (Hoe). (C) Bar chart showing the number of PRV^+^ neurons in the T9, T10, and T11 areas of the four groups. Values represent the mean ±SD. *n* = 5/group. *p < 0.05, compared with the GS group, ^#^ *p* < 0.05, compared with the GS+EA group, and ^&^ *p* < 0.05, compared with the MN group by one‐way ANOVA with LSD‐t. Green fluorescent protein (GFP, green), PRV (red), microtubule‐associated protein (Map2, white), and Hoe (blue). Scale bars =50 µm in (B1)–(B14), (B17)–(B20), (B23), and (B24); 10 µm in (B15) and (B16), (B21), and (B22). GS: gelatin sponge scaffold with no cells; GS+EA: GS combined electroacupuncture; MN: MSC‐derived neural network; MN+EA: MN combined with electroacupuncture

To explore whether EA promoted the integration of transplanted MSC‐derived neuron‐like cells with the host spinal cord neural circuit, PRV was injected into the sciatic nerves bilaterally, allowing for retrograde trans‐synaptic tracing (Figure 4A). The distribution and frequency of PRV‐infected neurons at the injury/graft site (T10) and the areas rostral (T9) and caudal (T11) to the injury/graft site 8 weeks after implantation were examined (Figure 4B). PRV‐labeled neurons were not detected in the rostral (T9) areas of the GS (Figure 4B1 and B2) or GS+EA (Figure 4B7,B8) groups. However, the number of PRV‐labeled neuron‐like cells at the injury/graft (T9) site in the MN+EA group (Figure 4B21,B22,C; 6.80 ± 1.20) was significantly increased compared with that in the MN group (Figure 4B15,B16,C; 2.50 ± 1.20, *p* < 0.05). PRV‐labeled neuron‐like cells were absent or not detected at the injury/graft (T9) sites of the GS (Figure 4B3,B4) and GS+EA (Figure 4B9,B10) groups. In the area rostral (T9) to the graft region, more PRV‐labeled neurons were identified in the MN+EA group (Figure 4B19,B20,C; 7.10 ± 0.50) than in the MN group (Figure 4B13,B14,C; 4.30 ± 0.60, *p* < 0.05). In areas caudal (T11) to the injury/graft site, the number of PRV‐labeled neurons in the MN+EA group (Figure 4B23,B24,C; 27.10 ± 1.10) was significantly increased compared with those in the GS (Figure 4B5,B6,C; 19.03 ± 2.54, *p* < 0.05) and GS + EA (Figure 4B11,B12,C; 22.80 ± 1.60, *p* < 0.05) groups. However, the number of PRV‐labeled neurons in areas caudal (T11) to the injury/graft site did not differ significantly between the MN+EA and MN (Figure 4B17,B18,C; 26.40 ± 0.80, *p* > 0.05) groups. These results indicated that EA treatment facilitated the integration of transplanted MSC‐derived neuron‐like cells into the host spinal cord neural circuit, which may ultimately serve as a neuronal relay able to transmit neural signals from the rostral host neurons to the grafted MN, allowing signals to reach the area caudal to the graft region of the spinal cord.

### 3.6 MN implantation combined with EA attenuates local inflammation

Inflammatory reactions commonly result in a cascade of secondary injuries after SCI. To assess inflammation 8 weeks after SCI, CD68‐positive macrophage/microglia were assessed (Figure S5). Immunofluorescence staining revealed fewer CD68‐reactive cells in the injury/graft site of the spinal cord in the MN+EA group compared with those in the MN, GS+EA, and GS groups (Figure S5A–D), which is consistent with the results of Western blot analysis (Figure S5E,F). These results suggested that implantation of MN, combined with EA, may have beneficial effects associated with the suppression of local inflammatory reactions following SCI.

### 3.7 Regeneration of 5‐Hydroxytryptamine (5‐HT)‐positive axons

Axonal regeneration was assessed by immunohistochemistry. Although 5‐HT‐positive axons reached the rostral host/injury interface (Figure S6A,A1), none of them crossed the rostral host/injury interface into the injury/graft site in the GS group (Figure S6A,A2). However, in the GS+EA and MN groups, some 5‐HT positive axons appeared to extend into the graft site but did not reach the caudal injury/host interface (Figure S6B,B3,C,C3). Remarkably, in the MN+EA group, significantly more 5‐HT positive axons regenerated into the injury/graft site (Figure S6D2 compared with those in the other groups (Figure S6E, *p* < 0.05). These results suggested that MN+EA could promote 5‐HT‐positive axon regeneration into the graft site.

### 3.8 Improvements in paralyzed hindlimb motor function

**FIGURE 5:**
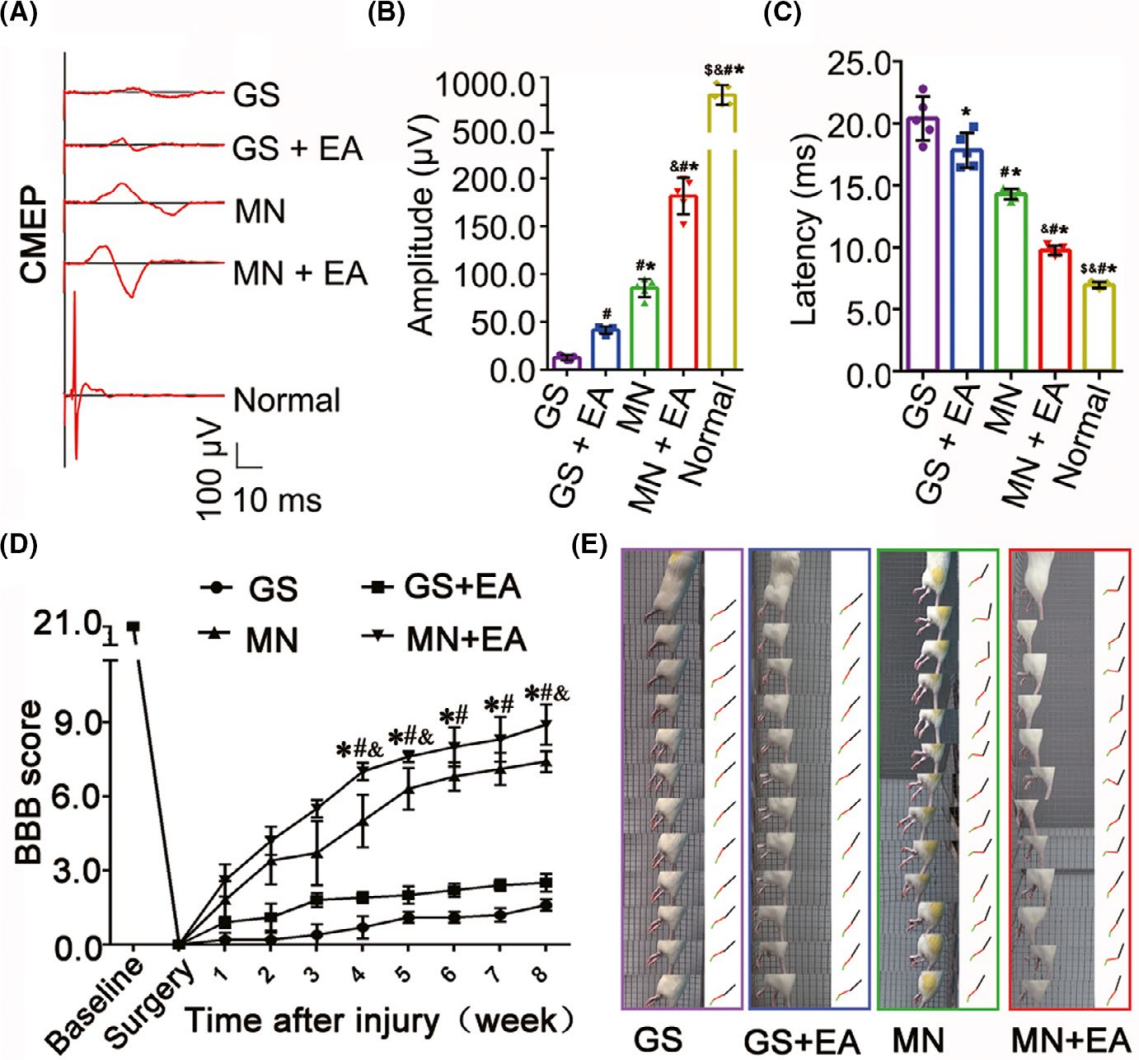
Outcomes of the BBB locomotion assessment, grid climb test, and electrophysiology. (A) Cortical motor‐evoked potentials (CMEPs) were obtained by electrophysiological analysis in the GS, GS+EA, MN, and MN+EA groups. (B, C) Bar charts of the amplitude (B) and latency (C) of CMEPs, showing the higher amplitudes and shorter latencies of the CMEPs in the MN+EA group compared with the GS (*p < 0.05), GS+EA (^#^ *p* < 0.05), and MN (^&^ *p* < 0.05) groups. Values represent the mean ±SD (*n* = 5/group; one‐way ANOVA with LSD‐*t*). (D) Comparison of Basso, Beattie, and Bresnahan (BBB) score for hindlimb locomotor function in the GS, GS+EA, MN, and MN+EA groups. Values represent the mean ± SD (*n* = 10/group; one‐way ANOVA). *p < 0.05 compared with the GS group, ^#^ *p* < 0.05 compared with the GS+EA group, and ^&^ *p* < 0.05 compared with the MN group, by one‐way ANOVA. (E) Grid climb test was performed in the GS, GS+EA, MN, and MN+EA groups at 8 wpi. GS: gelatin sponge scaffold with no cells; GS+EA: GS combined electroacupuncture; MN: MSC‐derived neural network; MN+EA: MN combined with electroacupuncture

Behavioral observations, electrophysiological detection, the BBB open‐field motor assessment, and modified grid climbing tests were then performed to assess the functional recovery of rats subjected to the various treatments. In normal animals, stimulation at certain points of the sensorimotor cortex could evoke CMEPs with short latencies and large response amplitudes (Figure 5A–C). Small CMEPs could be evoked in the GS group (Figure 5A). In the GS+EA, MN, and MN+EA groups, EA treatment or MN transplantation markedly improved CMEP performance compared with that in the GS group (Figure 5B,C, *p* < 0.05), and MN+EA treatment further shortened the latency and increased the amplitudes of CMEPs (Figure 5B,C, *p* < 0.05). BBB open‐field motor assessments showed that the hindlimbs of all rats were completely paralyzed following T10 spinal cord transection. Over 8 weeks after operation and transplantation, hindlimb locomotor performance showed progressive improvements in all groups. At 8 wpi, the mean BBB score in the MN+EA group was 8.92 ± 0.81. Some animals in the MN+EA group showed the extensive movement of all three hindlimb joints and interval plantar support of the paw, with BBB scores as high as 10 (Figure 5D). At 8 wpi, the 45° sloping grid climbing method was used to evaluate the spontaneous placement reflex triggered by direct touch (Figure 5E; Video S1 shows the grid climb test in the GS, GS+EA, MN, and MN+EA groups). The hindlimbs of rats in the GS group were dragged behind when rats climbed the sloping grid with their forelimbs. In the GS+EA and MN groups, the rats climbed up the grid using the backs of their paws. The rats in the MN+EA group, however, stepped on the grid with the plantar surfaces of their hind feet and displayed the coordinated movement of their forelegs and hindlegs.

### 3.9 EA increases NT‐3 levels in the injured spinal cord

**FIGURE 6:**
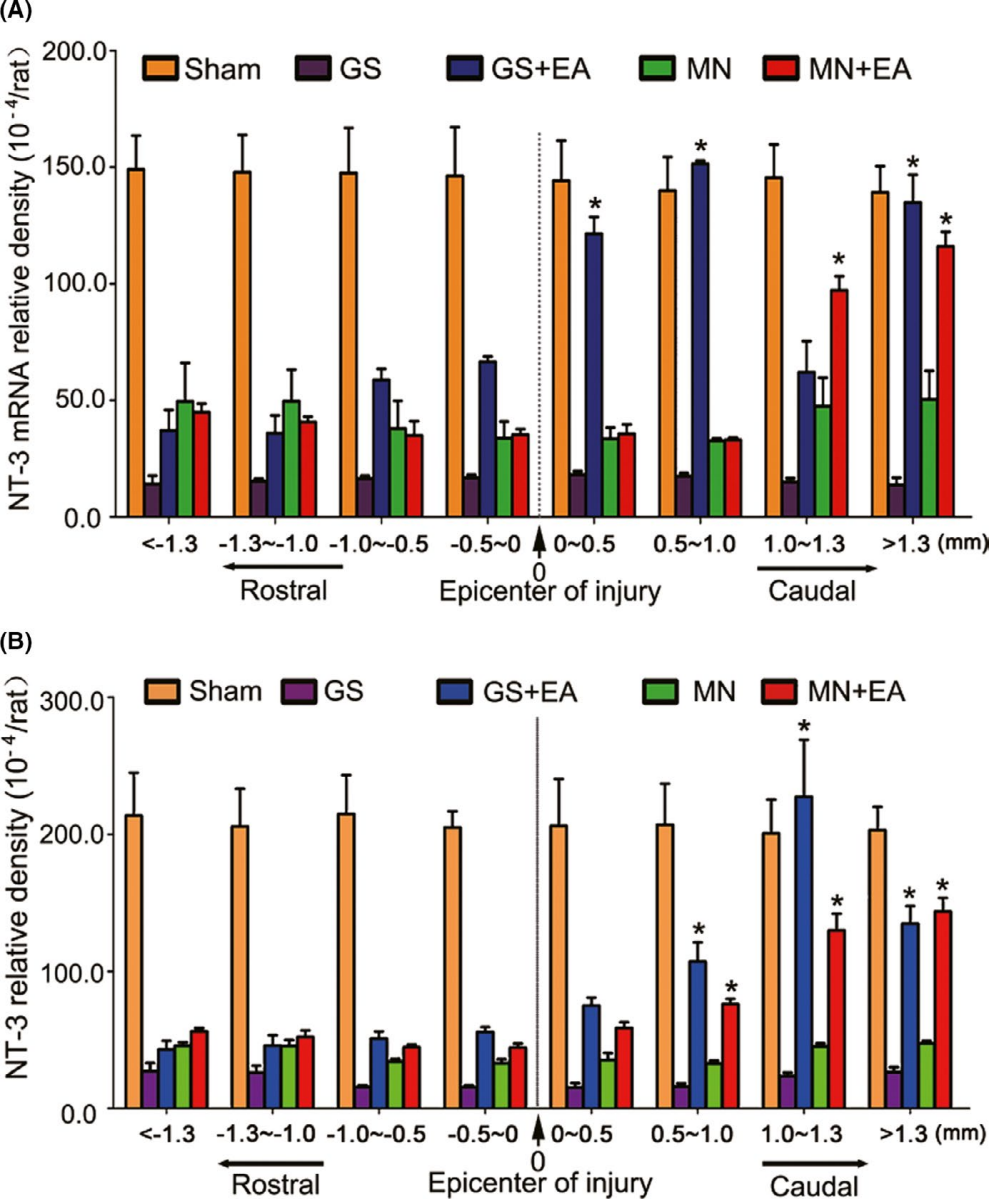
The density of neurotrophin‐3 (NT‐3) mRNA and protein in the injured spinal cord. (A) Bar charts displaying the relative densities of NT‐3 mRNA in eight regions in the rostral and caudal areas relative to the injury site of the spinal cord for five groups. The density of NT‐3 mRNA was significantly increased in the caudal site compared with the rostral area in the GS+EA and MN+EA groups. Values represent the mean ±SD (*n* = 5/group, *p < 0.05). (B) Bar charts showing the relative densities of NT‐3 protein expression in eight regions in the rostral and caudal areas relative to the injury site of the spinal cord for five groups. The density of NT‐3 protein was significantly increased in the caudal area compared with the rostral area in the GS+EA and MN+EA groups. Values represent the mean ±SD (*n* = 5/group, *p < 0.05). GS: gelatin sponge scaffold with no cells; GS+EA: GS combined electroacupuncture; MN: MSC‐derived neural network; MN+EA: MN combined with electroacupuncture

To evaluate NT‐3 expression in the injured spinal cord following various treatments, the injury/graft site and adjacent segment tissues were processed for immunofluorescence and *in situ* hybridization stainings. NT‐3 levels were calculated by measuring the standardized density of NT‐3 immunofluorescence and *in situ* hybridization stainings within the defined anatomical regions sampled from the Sham, GS, GS+EA, MN, and MN+EA groups. *In situ* hybridization staining showed that the relative density of NT‐3 mRNA‐positive staining among eight regions of the injured spinal cord in the Sham group at 2 wpi was not significantly different comparing with each other (Figures 6A and S7A,A1–A3). Compared with the Sham group, the relative density of NT‐3 mRNA‐positive declined in all eight regions, especially within the injury/graft site, in the GS and MN groups (Figures 6A and S7B,B1–B3,D,D1–D3). However, the relative density of NT‐3 mRNA‐positive staining in the GS+EA and MN+EA groups significantly increased compared with corresponding part in the GS and MN groups (Figures 6A and S7C,C1–C3,E and E1–E3). The GS+EA group frequently exhibited dense clusters of NT‐3‐mRNA‐positive staining in regions (0–0.5 mm, 0.5–1.0 mm, 1.0–1.3 mm, and >1.3 mm) adjacent to the caudal injury epicenter (Figures 6A and S7C), whereas the MN+EA group only exhibited clusters in areas (1.0–1.3 mm and >1.3 mm) adjacent to the caudal injury site (Figures 6A and S7E). NT‐3 immunofluorescence staining showed that the relative densities of NT‐3 immunofluorescence in the MN+EA and GS+EA groups were significantly increased compared with those in the GS and MN groups in the regions 1–1.3 mm and >1.3 mm adjacent to the caudal injury site (Figures 6B and S8). The increase in the relative density of NT‐3‐positive staining in the caudal regions 1–1.3 mm relative to the epicenter was the most pronounced in the GS+EA group. In the MN+EA group, the density of NT‐3‐positive staining in the regions 1–1.3 mm and >1.3 mm caudal to the epicenter was significantly higher than that in the regions −1.3–1 mm and < −1.3 mm adjacent to the rostral injury site (Figures 6B and S8E,E1–E3).

### 3.10 Transwell migration of MSCs

We explored the effects of NT‐3 on the migration capability of TrkC‐MSCs *in vitro*. As shown in Figure S9, compared with the control group (0 ng/ml NT‐3), the average number of migrated TrkC‐MSCs progressively increased in a dose‐dependent manner with increasing NT‐3 concentration, peaking at 40 ng/ml NT‐3. Furthermore, the effects of NT‐3 on the induction of TrkC‐MSC migration was inhibited upon pre‐treatment with K252a (Figure S10).

## 4 DISCUSSION

This study has demonstrated the disease‐modifying capabilities of an implanted MN combined with EA treatment in a rat spinal cord transection model. The results showed that combined with EA treatment, MSC‐derived neuron‐like cells initially seeded on the MN showed improved survival rate, but more importantly, they ultimately migrated and differentiated into a neuron‐like phenotype in the injured cord. The neuron‐like cells derived from the MSCs could serve as the structural and functional relaying elements for restoring the injured neural circuit. In addition, we showed that EA treatment could effectively modulate the microenvironment of the injured area, especially at the caudal border, by increasing the production of endogenous NT‐3. Experimental evidence indicated that NT‐3 is instrumental for the enhanced integration of the MN into the local neural circuitry, providing a structural basis for effective signal transduction across the injured spinal cord. More strikingly, we have shown that MN+EA treatment improved the motor function of the paralyzed hindlimb, as evaluated by BBB scores.

The original design of this study was to use EA treatment to improve the integration of the implanted MN with the host neural circuits. The results first confirmed the formation of synapse‐like structures between the host descending 5‐HT axons, propriospinal neurons, and the MSC‐derived neuron‐like cells. Next, the MSC‐derived neuron‐like cells were found to have the potential to synthesize common neurotransmitters, such as ChAT, CaMKII, and GABA, suggesting that the transplanted MN comprises both inhibitory and excitatory donor neuron‐like cells. Interestingly, synapse‐like structures were also identified between some of the MSC‐derived neuron‐like cells. Occasional MSC‐derived neuron‐like cells were observed to form synapse‐like contacts with the host neurons in the area caudal to the injury/graft site, as demonstrated by IEM, suggesting that these structural components may form a potential neuronal relay.^29^, ^30^ These structures included (1) synapse‐like structures between the regenerating host descending axons from the area rostral to the injury/graft site and donor neuron‐like cells in the injury/graft site of the spinal cord; (2) donor neuron‐like cells that produced neurotransmitters; (3) long extending processes from the donor neuron‐like cells to the area caudal to the injury/graft site; and (4) synapse‐like structures between the long extending processes that emanate from the donor neuron‐like cells and the host neurons in the area caudal to the injury/graft site. The integration of MSC‐derived neuron‐like cells with host neuronal circuits was further verified using a PRV trans‐synapse retrograde tracing study, which showed that in the MN+EA group, more donor neuron‐like cells in the injury/graft site and more host neurons in the area rostral to the injury/graft site were labeled by PRV. These results indicated that the implanted MN established structural contacts and functional connections with the host ascending and descending axons.

In the present study, we have noted the wider occurrence of mature MSC‐derived neuron‐like cells after EA treatment. However, in our previous study, without the use of EA,^8^ the majority of MSC‐derived donor cells existed as immature neurons 2 months after implantation. A similar feature was also reported by other authors using exogenous neuronal relays for the functional repair of SCI. This phenomenon may be associated with the slow process of donor neuron maturation and the dwindling of donor cells over time.^30^ For example, human spinal cord‐derived neural progenitor cells generated a high proportion of naïve neurons in the injured spinal cord of rhesus monkeys 9 months after implantation. Human H9 NSCs, when grafted into SCI sites in immunodeficient rats, first expressed markers of neuronal maturity and continued maturation over a period of 1.5 years.^31^

We believe that a cell therapy regimen that combines a bioscaffold‐based cell delivery method with EA treatment, as was adopted in this study, can greatly improve donor cell survival in the hostile SCI microenvironment.^9^ Using this strategy, the donor cell population showed a better survival rate and maintained a neuron‐like phenotype for at least eight weeks after EA. Another finding from this study was the importance of the NT‐3 level in the injured spinal cord, which was markedly augmented by EA treatment.^11^, ^25^ Our recent study has also verified that EA could stimulate the spinal nerve branches of the dorsal root ganglion (DRG) cells to increase the release of calcitonin gene‐related peptide (CGRP) in the injured spinal cord, and then trigger the synthesis and secretion of NT‐3 in spinal neurons by activating the CGRP/RAMP1/αCaMKII pathway and the intrinsic growth ability of spinal neurons after SCI.^32^ Additionally, NT‐3 can promote the survival and differentiation of neurons necessitated for the development of descending motoneuron pathways.^33^

Studies have demonstrated that NT‐3 can attenuate neuroinflammation.^34^, ^35^ It is well documented that vigorous inflammatory reaction occurs readily following the SCI. Appropriate inflammatory response has beneficial effects notably in the early stage of SCI, such as the removal of cellular debris through phagocytosis. However, excessive and prolonged inflammatory response can cause damage to the surrounding tissues and is unfavorable for the spontaneous regeneration and functional recovery.^36^ Several studies have reported that EA or MSC transplantation can effectively regulate the inflammatory responses; more importantly, it protects the injured spinal cord from the secondary pathological reaction. The therapeutic approaches include the EA, cell therapies, monoclonal antibodies among others.^37^, ^38^, ^39^ It has been reported that EA can reduce inflammation via exciting specific neural pathways. This would further activate particular receptors in the spleen that suppressed pro‐inflammatory molecules.^37^ In addition, MSCs can reverse the phenotype of M1 macrophage/microglia towards anti‐inflammatory M2 subpopulation *in vitro* and *in vivo*.^39^ In this study, we found that the combination of EA and MN could reduce the expression of CD68 protein in the injured spinal cord at 8 weeks SCI. The above when taken together with the present results suggest that EA in combination with MN transplantation can exert a synergistic effect by inhibiting the inflammatory response in SCI.

Very interestingly, in a separate recent study, it has been reported that MSC transplantion downregulates the NT‐3 expression level in ischemia‐injured brain.^40^ The discrepancy in results may be attributed to differences in phenotype of transplanted MSCs used and also the use of different animal models. Further studies are clearly desirable to decipher the underlying mechanisms. Besides the neurotrophin and immune regulation,^41^ emerging evidence has shown that MSC transplantion can protect and improve the neuron function after injury via exosomes^42^ and mitochondrial transfer.^43^ Notwithstanding of the above, it is suggested that MN+EA treatment as demonstrated in the present study can repair the SCI effectively which is likely through multiple effects. All in all, it is suggested that EA can facilitate the cell survival, migration, differentiation, and integration of transplanted MNs with the host spinal cord, resulting in functional improvements of the hindlimbs. The present results indicated that EA acts through the enhancement of local NT‐3 production, improving the microenvironment of the injured spinal cord and promoting the reconstruction of neural circuits involving *TrkC*‐modified MN transplant and host neurons.

In summary, we showed that MN transplantation, when combined with EA application, can facilitate the survival, migration, and maintenance of neuronal characteristics of MSC‐derived neuron‐like cells. MN+EA also promoted axonal regeneration and the integration of MSC‐derived neuron‐like cells with host neurons. Through the increased local production of NT‐3, EA can foster the biological functions of MSC‐derived neuron‐like cells. NT‐3 can improve the hostile microenvironment of the injured spinal cord by dampening local inflammation, which is crucial for the reconstruction of neural circuitry.

## CONFLICT OF INTEREST

The authors have no financial conflicts of interest.

## AUTHORS’ CONTRIBUTIONS

YSZ and YD designed and supervised the study. YY, HYX, DQW, GHW, LJW, HJ, JWR, BJ, and YQW performed the experiments and collected the data. YY, HYX, YD, and YSZ summarized, analyzed, and plotted the data, and drafted the manuscript. YD, YSZ, and XZ wrote and finalized the manuscript. All authors have given approval to the final version of the manuscript.

## Supporting information

Supplementary Material

Video S1

Supplementary Material

## Data Availability

The data that support the findings of this study are available from the corresponding author upon reasonable request.
