# Supplementary material for: Electroacupuncture facilitates the integration of a grafted TrkC‐modified mesenchymal stem cell‐derived neural network into transected spinal cord in rats via increasing neurotrophin‐3
Source: CNS Neurosci Ther. 2021 Mar 24;27(7):776–91. doi: 10.1111/cns.13638 (PMC8193704; doi:10.1111/cns.13638)
Supplement: Supplementary file 1 — Supplementary Material [file CNS-27-776-s003.pdf]

Supporting Information

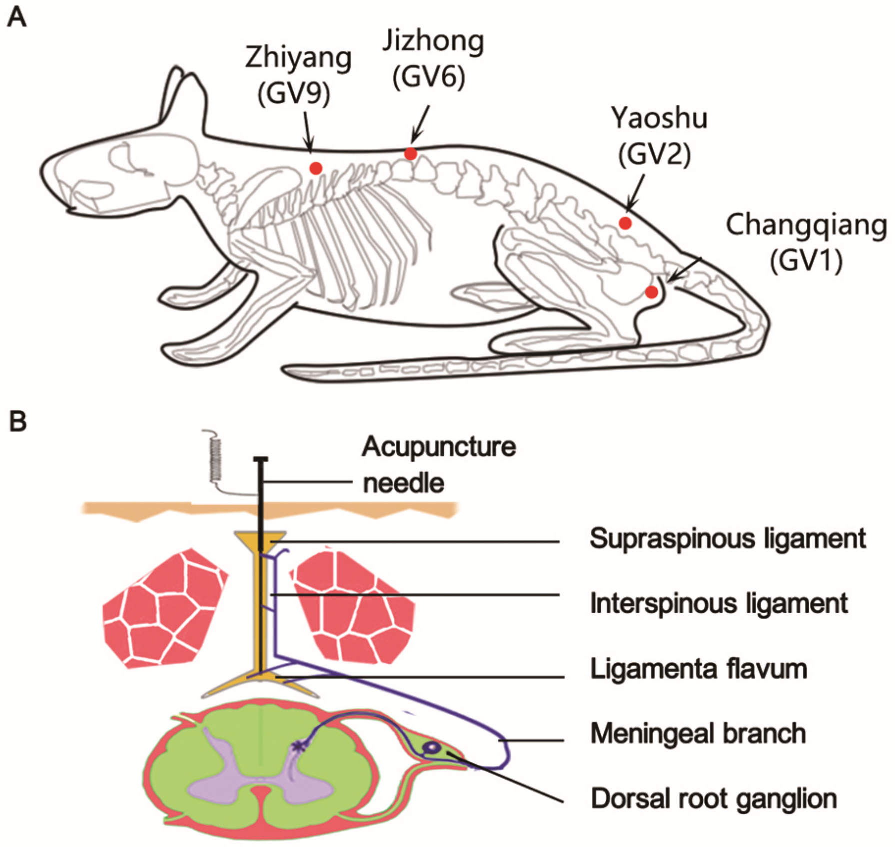

FIGURE S1 Showing two pattern diagrams of the location of the GV-EA. (A) A pattern diagram indicates selected electroacupuncture acupoints located at GV in rat. Arrows show the four GV acupoints: Zhiyang (GV9), Jizhong (GV6), Yaoshu (GV2) and Changqiang (GV1). (B) Another pattern diagram of the afferent pathway of GV-EA stimulation and the figure indicates that the stimulation information of GV-EA might have been transmitted first to the dorsal root ganglion (DRG) by peripheral branches of meningeal branch and finally transmitted to the spinal cord by the DRG's central branches.

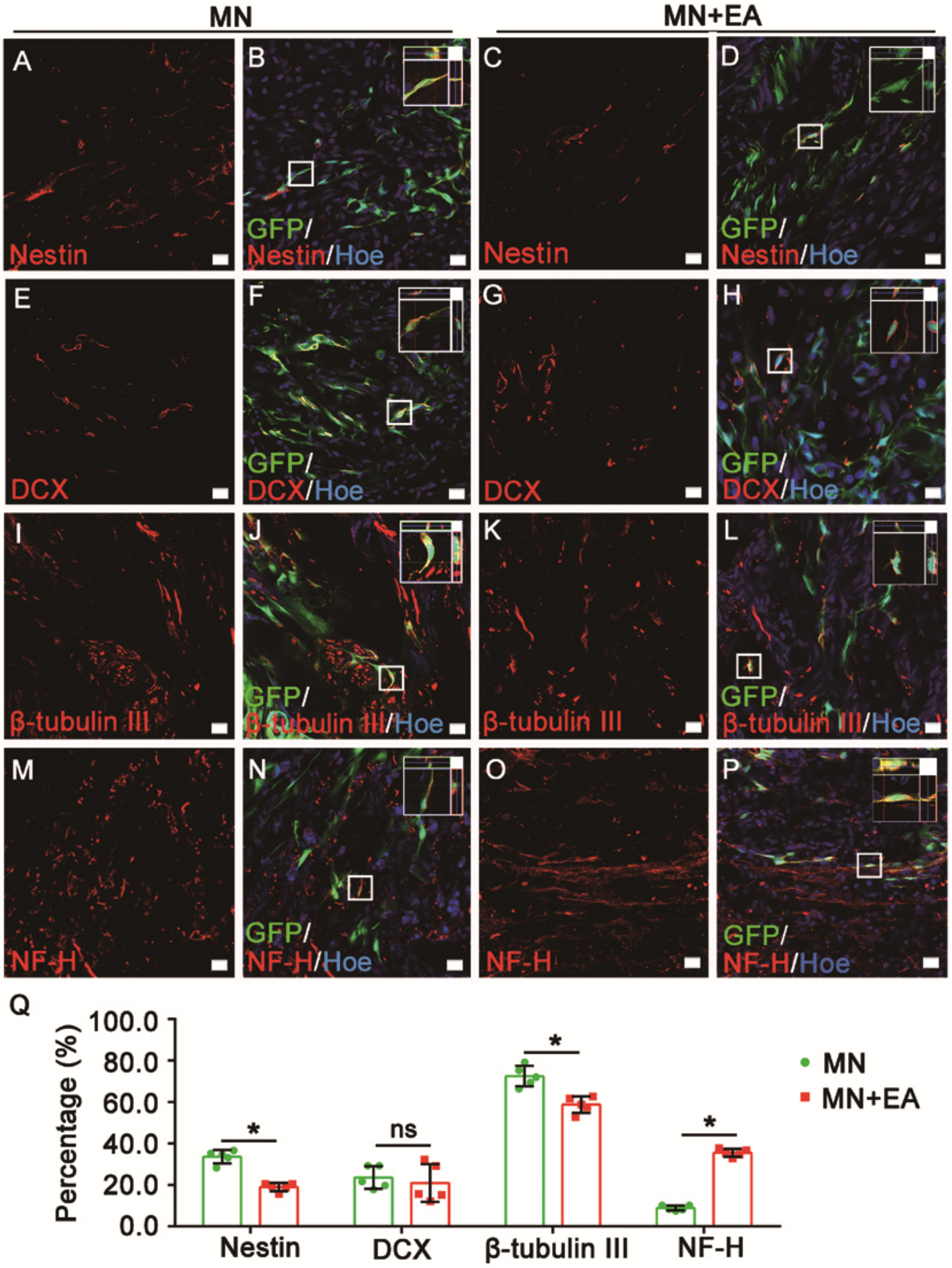

FIGURE S2 Donor mesenchymal stem cells (MSCs) increased the rate of differentiation into neuron-like cells at the injury/graft site of the spinal cord following electroacupuncture (EA) treatment. (A–P) MSCs differentiated into Nestin-positive neural stem cells (A)–(D), doublecortin (DCX)-positive neural progenitor cells (E)–(H), β-tubulin III-positive immature neuron-like cells (I)–(L), and neurofilament-H (NF-H)-positive neuron-like cells (M)–(P) after 8 weeks of transplantation in the MSC-derived neural network (MN) and MN+EA groups. Inset shows the co-localization between green fluorescent protein (GFP), Hoechst33342

(Hoe), and Nestin (B) and (D), DCX (F) and (H), β-tubulin III (J) and (L) or NF-H (N) and (P). Scale bars = 20 μm in (A)–(P). (Q) Quantification of the percentages of Nestin-, DCX-, β-tubulin III-, and NF-H-positive cells among all MSC-derived cells in the MN and MN+EA groups. Values represent the mean ± SD (*n* = 5/group, Student's t-test, *$p < 0.05$).

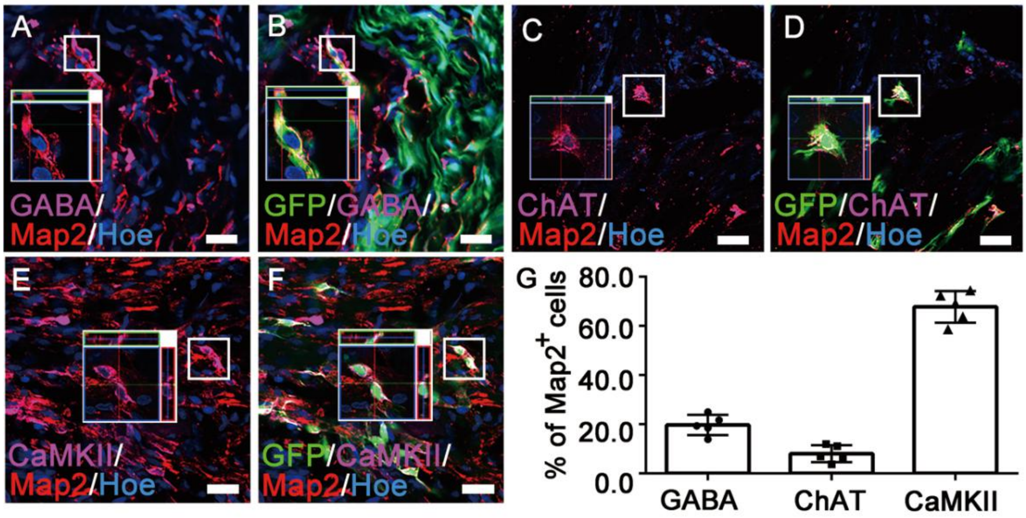

FIGURE S3 Phenotypic characterization of MSC-derived neuron-like cells in the MN+EA group at 8 weeks after transplantation. (A)-(F) Confocal images of neural progenitor cell grafts. Map2 expressing neuron-like cells in grafts express GABA (A) and (B), ChAT (C) and (D), and CaMKII (E) and (F). Insets show co-localization of GFP, Map2 and Hoe with GABA (B), ChAT (D) or CaMKII (F). (G) Bar chart showing the quantification of neuronal subtypes in the MN+EA group. After 8 weeks, most MSC-derived neurons express excitatory neuron markers CaMKII. Values represent mean ± SD. *n* = 5/group. Scale bars = 20 μm in (A)-(F).

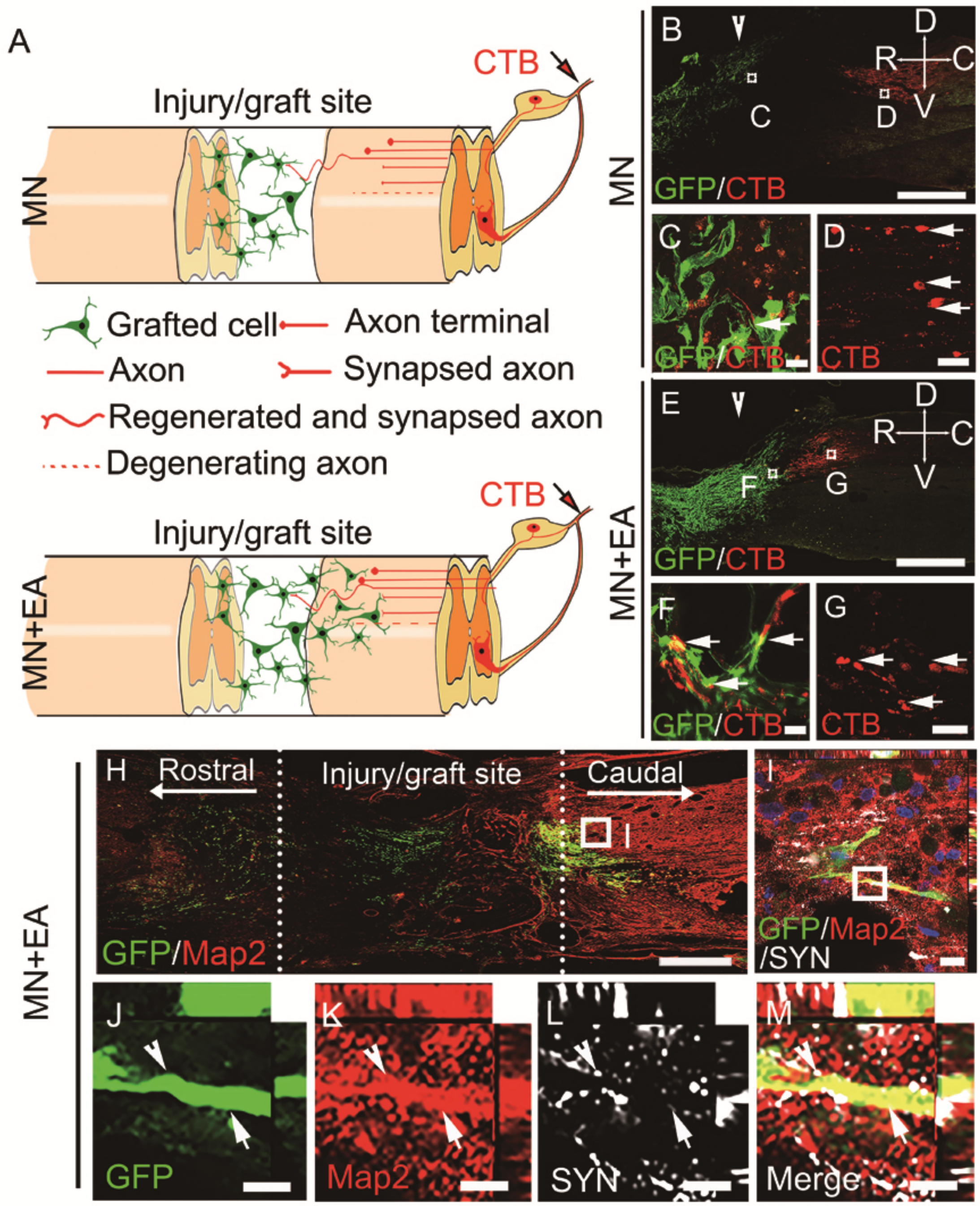

FIGURE S4 Implanted MSC-derived cells migrated into the caudal spinal cord and made contacts with host caudal neurons. (A) A schematic diagram depicting the labeling of ascending nerve fibers by injecting CTB-555 into the sciatic nerve. (B) and (E) Low-magnification images showing GFP$^+$ cells (green) and CTB$^+$ ascending nerve fibers (red) in sagittal sections of spinal cord; arrowheads indicate the interface between the injury/graft site and the caudal host tissue, respectively. (C) and (F) Sagittal section of injured spinal cord showing CTB$^+$ ascending nerve fibers making potential synaptic contacts (arrows) with GFP$^+$ cells. (D) and (G) Sagittal view of injured spinal cord showing CTB$^+$ ascending nerve fiber terminals (arrows). (H) A sagittal section showing GFP$^+$ MSC-derived neuron-like cells migrated into the host caudal spinal cord tissue and contacted with Map2$^+$ nerve fibers (I). (J)-(M) Showing

enlarged view of images of boxed area in (I), triple-labeled with anti-GFP (J), Map2 (K) and SYN (L), and their merged image (M). Arrowheads indicate host Map2$^+$/SYN$^+$ axon terminals contacting with a GFP$^+$/Map2$^+$ MSC-derived neuron-like cell process. Arrows point at GFP$^+$/Map2$^+$ MSC-derived neuron-like cell process. D, V, R and C on the right corner in (B) and (E) denote dorsal, ventral, rostral, and caudal to the spinal cord, respectively. Scale bars = 1 mm in (B) and (E); 20 μm in (C)-(D); 500 μm in (H); 10 μm in (I); 5 μm in (J)-(M).

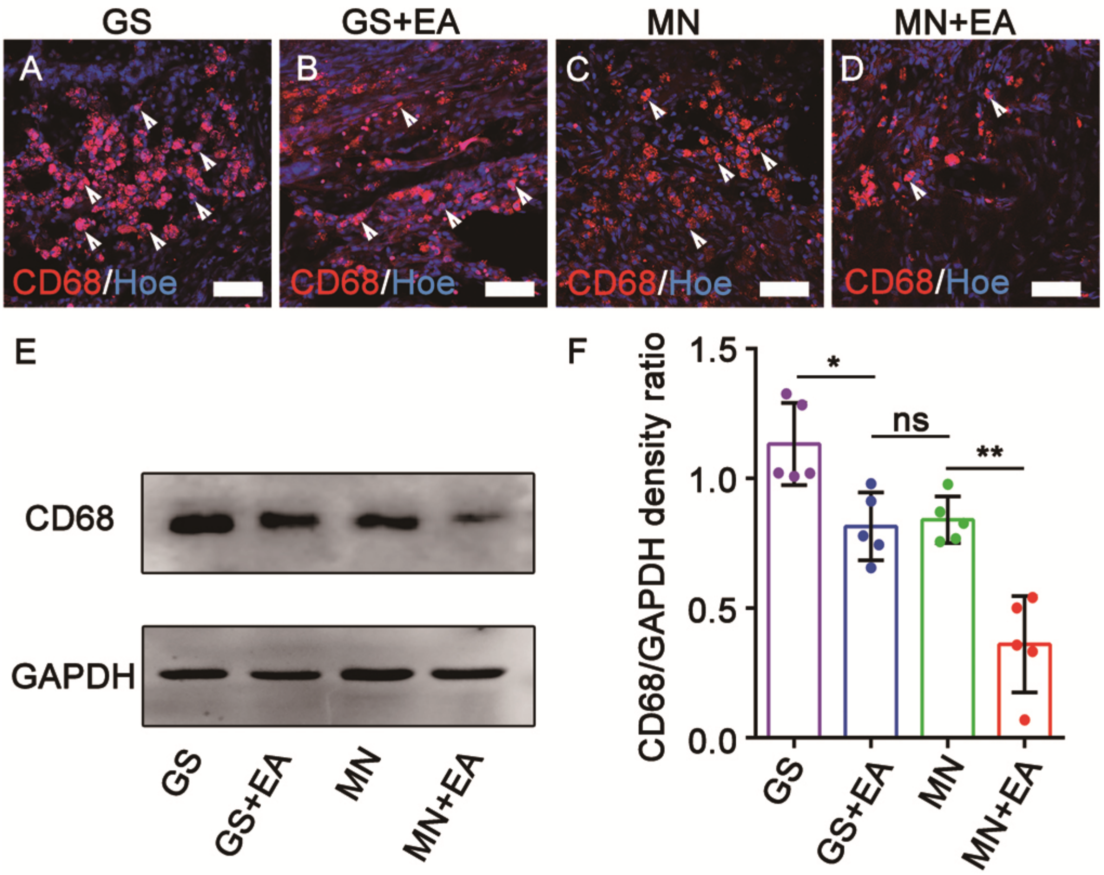

FIGURE S5 More effective inflammatory alleviation was observed after mesenchymal stem cell (MSC)-derived neural network (MN) implantation combined with electroacupuncture (EA) treatment. (A)–(D) $CD68^{+}$ cells at the injury/graft site of the spinal cord in the GS, GS+EA, MN, and MN+EA groups. Scale bars = 50 µm in (A)–(D). (E) Western blot analysis showing the protein levels of CD68 in each group. Glyceraldehyde 3-phosphate dehydrogenase (GAPDH) was used as a loading control. (F) Bar chart showing relative quantification of CD68 protein expression in all four groups. Values represent the mean ± SD. n =5/group. * and ** indicate $p < 0.05$ and $p < 0.01$, respectively. GS: gelatin sponge scaffold with no cells; GS+EA: GS combined electroacupuncture; MN: MSC-derived neural network; MN+EA: MN combined with electroacupuncture.

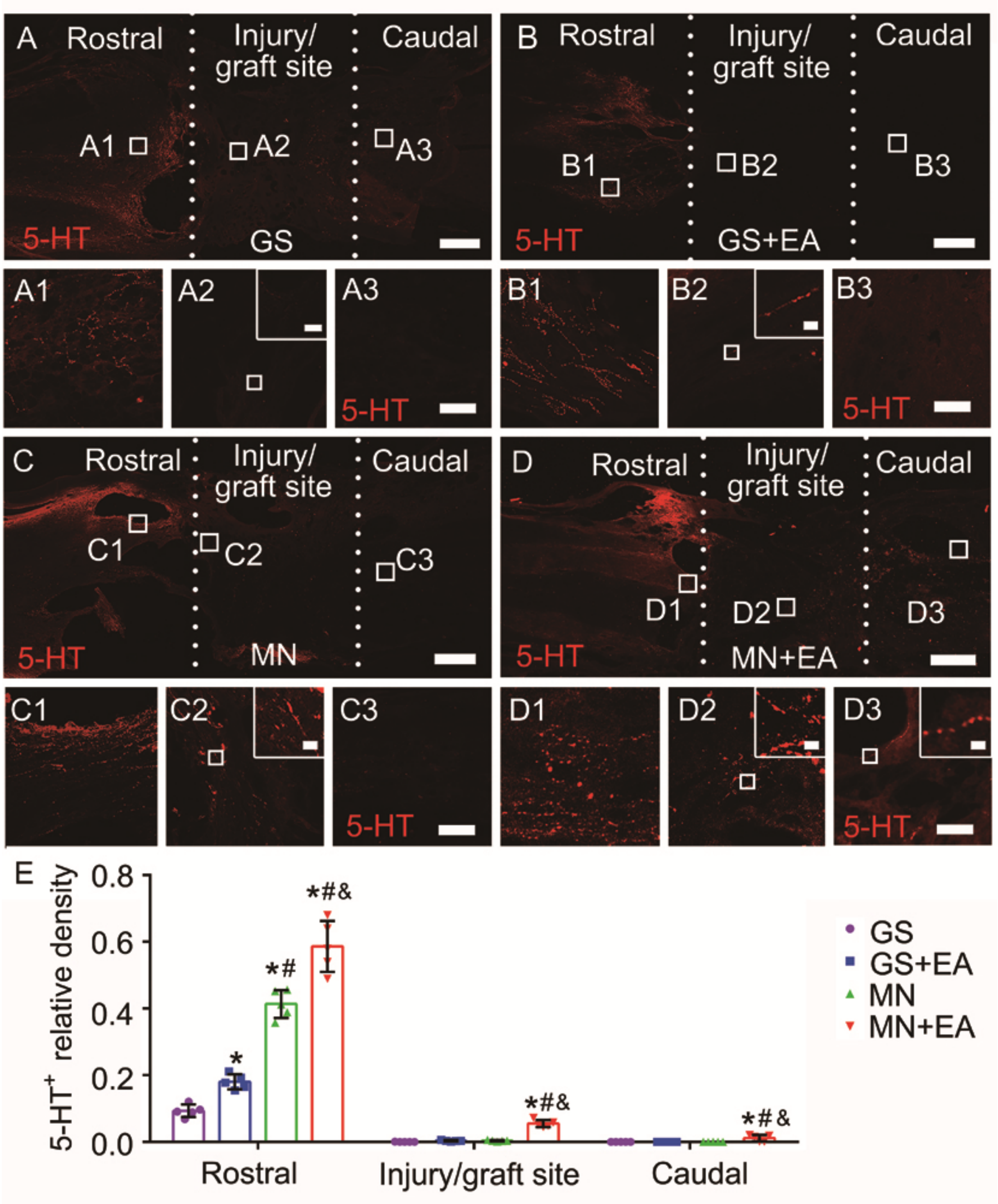

FIGURE S6 Regeneration of 5-hydroxytryptamine (5-HT)$^+$ axons. (A–D) Longitudinal sections of the spinal cord with 5-HT$^+$ axons in the GS (A), GS+EA (B), MN (C), and MN+EA (D) groups. (A1)–(D3) Immunofluorescent images of the boxes in (A)–(D), respectively. Scale bars = 500 μm in (A)–(D); 50 μm in (A1)–(D3); 10 μm in (F)–(I); 2 μm in (i). (E) Bar chart comparing the quantity of 5-HT$^+$ axons in the rostral and caudal areas relative to the injury/graft site among the four groups. Values represent the mean ± SD (n = 5/group, LSD-t, $^*p < 0.05$ *vs.* the GS group, $^\#p < 0.05$ *vs.* the GS+EA group, $^\&p < 0.05$ *vs.* the MN group). GS: gelatin sponge scaffold with no cells; GS+EA: GS combined electroacupuncture; MN: MSC-derived neural network; MN+EA: MN combined with electroacupuncture.

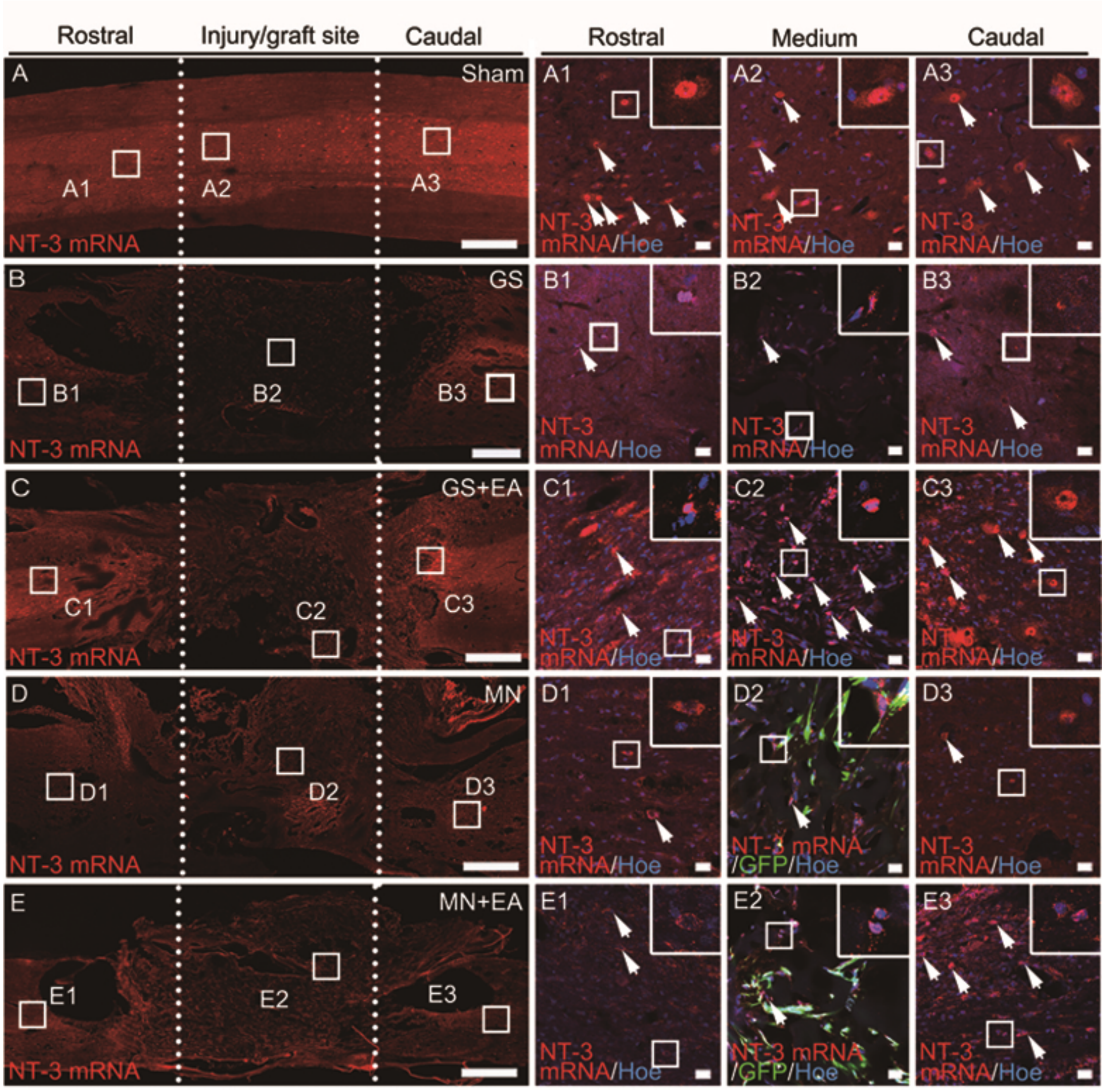

FIGURE S7 *In situ* hybridization analysis showing the distribution of NT-3 mRNA in injured spinal cord. (A)-(E3) Representative images showing the immunostaining of NT-3 mRNA within injured spinal cord in the Sham group (A), (A1)-(A3); GS group (B), (B1)-(B3); GS+EA group (C), (C1)-(C3); MN group (D), (D1)-(D3); and MN+EA group (E), (E1)-(E3), respectively. The cell nuclei were counterstained by Hoechst33342 (Hoe). The insets that showed a magnification (zoom) image of the cells in the tissue have been respectively added in (A)-(E3). Scale bars = 500 µm in (A)-(E); 10 µm in (A1)-(A3), (B1)-(B3), (C1)-(C3), (D1)-(D3) and (E1)-(E3).

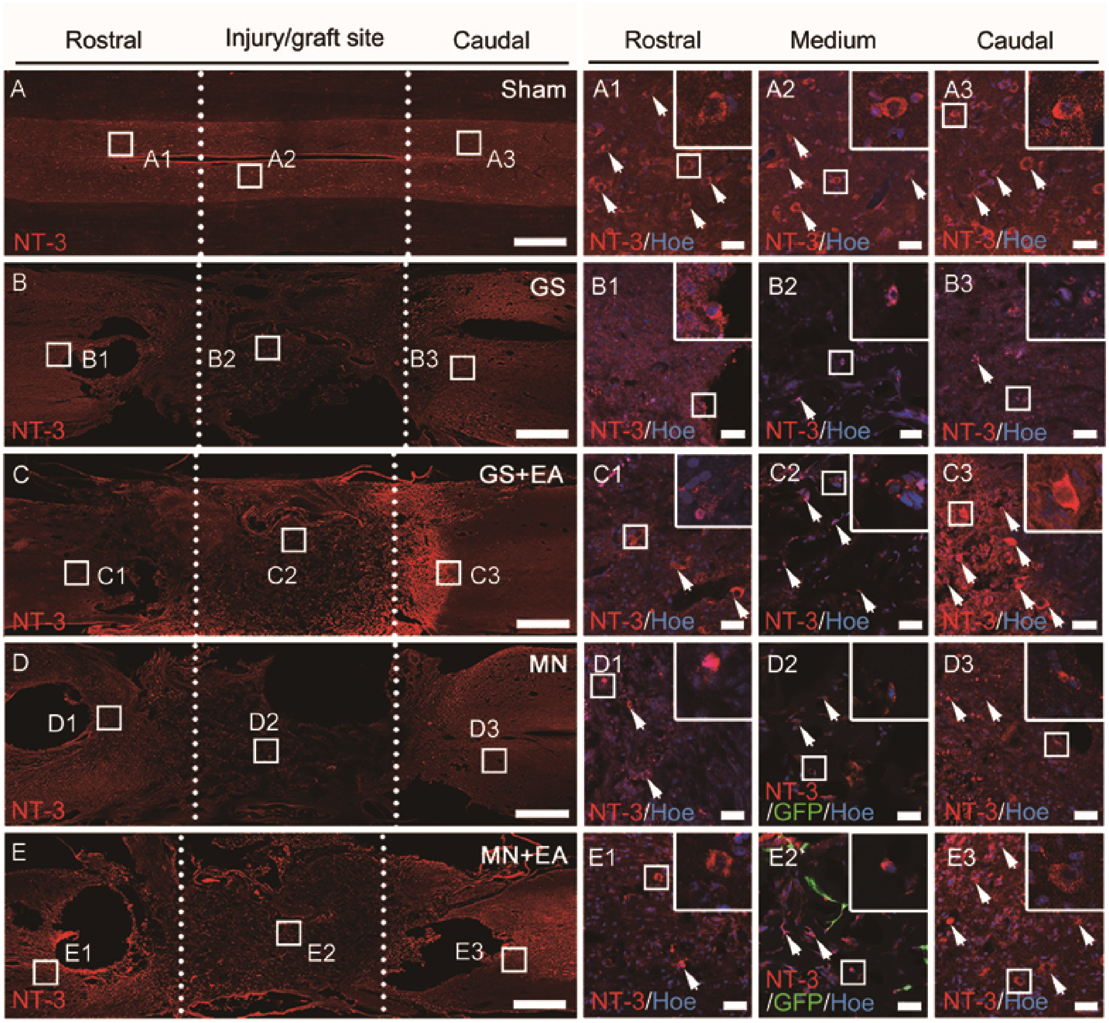

FIGURE S8 Distribution of NT-3 in injured spinal cord. (A)-(E3) Representative images showing NT-3 immunostaining in injured spinal cord in the Sham group (A), (A1)-(A3), the GS group (B), (B1)-(B3), the GS+EA group (C), (C1)-(C3), the MN group (D), (D1)-(D3) and the MN+EA group (E), (E1)-(E3), respectively. The cell nuclei were counterstained by Hoechst33342 (Hoe). The insets that showed a magnification (zoom) image of the cells in the tissue have been respectively added in (A)-(E3). Scale bars = 500 μm in (A)-(E); 20 μm in (A1)-(A3), (B1)-(B3), (C1)-(C3), (D1)-(D3) and (E1)-(E3).

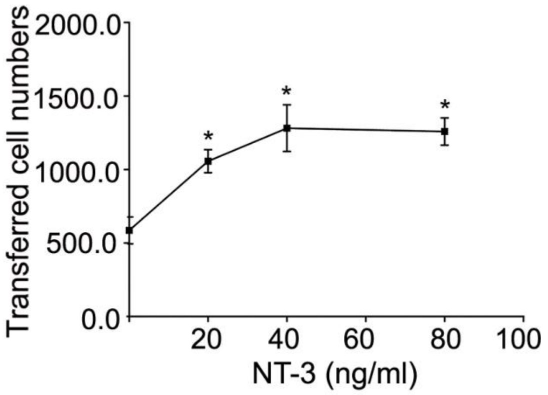

FIGURE S9 Effect of NT-3 on TrkC-MSC migration *in vitro* (48 h). NT-3 attracted TrkC-MSC migration in a dose-dependent manner. Results are presented as mean ± SD. *$p < 0.05$ *vs*. Control (NT-3 = 0 ng/ml).

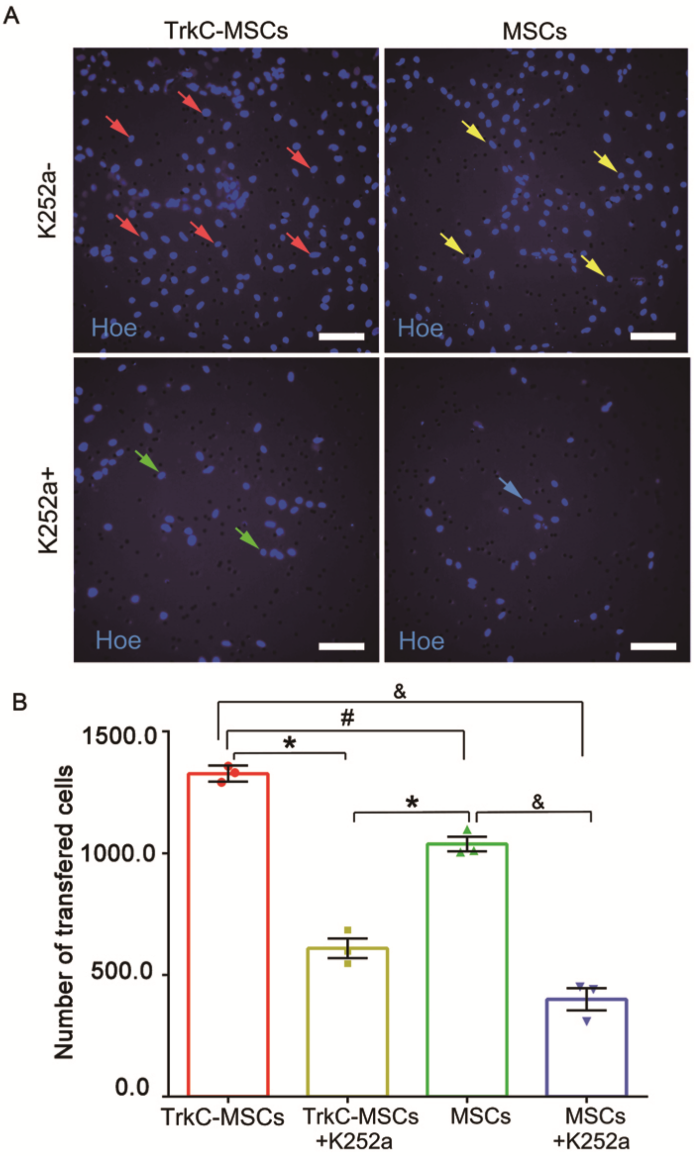

FIGURE S10 Chemotactic migration of TrkC-MSCs. (A) Transwell migration assay showed migration of MSCs and TrkC-MSCs in response to a solution of NT-3 ($c = 40$ ng/ml) with K252a ($c = 10$ nM) or a solution of NT-3 without K252a, respectively. The nuclei of MSCs and TrkC-MSCs were stained with Hoechst33342 (Hoe). Scale bars = 100 μm in (A). (B) Bar chart showing the number of migrating cells in different groups as assessed by Transwell experiment. Results are presented as mean ± S.D., $^{*}p < 0.05$ *vs.* TrkC-MSCs+K252a, $^{\#}p < 0.05$ *vs.* MSCs, $^{\&}p < 0.05$ *vs.* MSCs+K252a.

Table S1 Primary and secondary antibodies

Antibodies	Species	Type	Dilution	Source
Neurofilament 200 (NF)	Rabbit	Polyclonal IgG	1:400	Merck Millipore, Billerica, USA
Nestin	Mouse	Monoclonal IgG	1:200	Abcam, United Kingdom
Doublecortin (DCX)	Rabbit	Polyclonal IgG	1:400	Abcam, United Kingdom
β -tubulin III	Rabbit	Polyclonal IgG	1:500	Sigma, St. Louis, USA
Neurofilament 200 (NF-H)	Rabbit	Polyclonal IgG	1:600	Merck Millipore, Billerica, USA
Neurofilament 200 (NF-H)	Mouse	Monoclonal IgG	1:600	Sigma, St. Louis, USA
CD68	Mouse	Monoclonal IgG	1:100	R&D System
Postsynaptic density Protein 95 (PSD95)	Rabbit	Polyclonal IgG	1:800	Abcam, London, UK
Vesicular glutamate Transporter 2 (VGlut-2)	Rabbit	Polyclonal IgG	1:1000	Sigma, St. Louis, USA
Microtubule-associated protein 2 (MAP2)	Chicken	Monoclonal IgG	1:1500	Abcam, London, UK
Green fluorescent protein (GFP)	Rabbit	Polyclonal IgG	1:500	Merck Millipore, Billerica, USA
Green fluorescent protein (GFP)	Mouse	Monoclonal IgG	1:1000	Merck Millipore, Billerica, USA
Glyceraldehyde-3-phosphate dehydrogenase(GAPDH)	Mouse	Monoclonal IgG	1:2500	Proteintech, Rosemont, USA
calcium/calmodulin-dependent protein kinase (CaMKII)	Mouse	Monoclonal IgG	1:100	Santa Cruz Biotechnology, USA
γ-aminobutyric acid (GABA)	Rabbit	Polyclonal IgG	1:500	Boster, Wuhan, China
Microtubule-associated protein 2 (MAP2)	Mouse	Monoclonal IgG	1:1000	Sigma, St. Louis, USA
Microtubule-associated protein 2 (MAP2)	Rabbit	Polyclonal IgG	1:800	Sigma, St. Louis, USA
Choline acetyltransferase (ChAT)	Rabbit	Polyclonal IgG	1:800	Merck Millipore, Billerica, USA
Pseudorabies virus (PRV)	Rabbit	Polyclonal IgG	1:800	Abcam, London, UK
Synaptophysin (SYN)	Mouse	Monoclonal IgG	1:200	Sigma, St. Louis, USA
Neurotrophin-3 (NT-3)	Rabbit	Polyclonal IgG	1:300	Santa Cruz Biotechnology, Santa Cruz, USA
5-hydroxytryptamine (5-HT)	Rabbit	Polyclonal IgG	1:8000	Sigma, St. Louis, USA
glyceraldehyde-3-phosphate dehydrogenase (GAPDH)	Mouse	Monoclonal IgG	1:1000	Proteinteach, Rosemont, USA
Horseradish Peroxidase (HRP)	Rabbit	Polyclonal IgG	1:5000	Cell Signaling Technology, Boston, USA
Horseradish Peroxidase (HRP)	Mouse	Monoclonal IgG	1:2000	Cell Signaling Technology, Boston, USA
Streptavidin, Alexa Fluor™ 555 conjugate	---	---	1:500	Invitrogen, Waltham, USA
Hoechst33342 (Hoe)	---	---	1:1000	Beyotime, Shanghai, China
Alexa 647 conjuncted anti rabbit secondary antibody	Goat	Polyclonal IgG	1:500	Abcam, London, UK
Alexa555 conjuncted antirabbit secondary antibody	Goat	Polyclonal IgG	1:500	Abcam, London, UK
Alexa555 conjuncted antichickensecondary antibody	Goat	Polyclonal IgG	1:500	Invitrogen, Waltham, USA
Alexa647 conjuncted antichickensecondary antibody	Goat	Polyclonal IgG	1:500	Abcam, London, UK
Alexa488 conjuncted anti mouse secondary antibody	Goat	Polyclonal IgG	1:500	Invitrogen, Waltham, USA
Alexa555 conjuncted anti mouse secondary antibody	Goat	Polyclonal IgG	1:500	Abcam, London, UK
