# Supplementary material for: Electroacupuncture facilitates the integration of a grafted TrkC‐modified mesenchymal stem cell‐derived neural network into transected spinal cord in rats via increasing neurotrophin‐3
Source: CNS Neurosci Ther. 2021 Mar 24;27(7):776–91. doi: 10.1111/cns.13638 (PMC8193704; doi:10.1111/cns.13638)
Supplement: Supplementary file 3 — Supplementary Material [file CNS-27-776-s002.docx]

Supporting Information


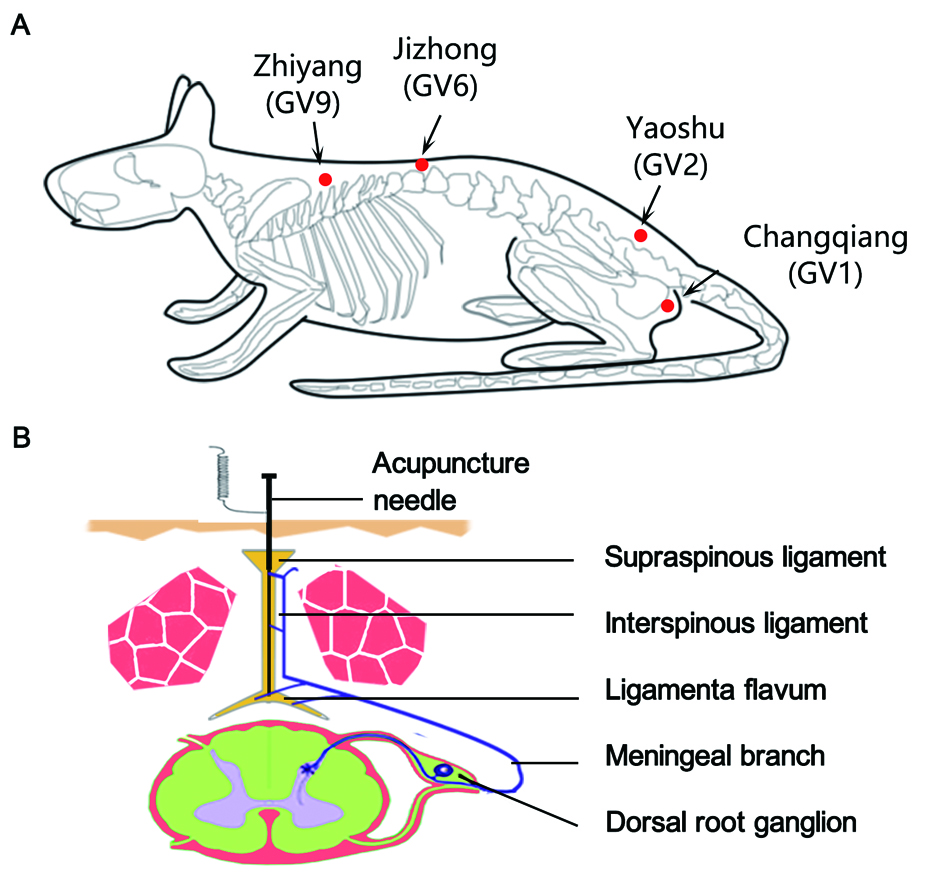


**FIGURE S1** Showing two pattern diagrams of the location of the GV-EA. (A) A pattern diagram indicates selected electroacupuncture acupoints located at GV in rat. Arrows show the four GV acupoints: Zhiyang (GV9), Jizhong (GV6), Yaoshu (GV2) and Changqiang (GV1). (B) Another pattern diagram of the afferent pathway of GV-EA stimulation and the figure indicates that the stimulation information of GV-EA might have been transmitted first to the dorsal root ganglion (DRG) by peripheral branches of meningeal branch and finally transmitted to the spinal cord by the DRG’s central branches.


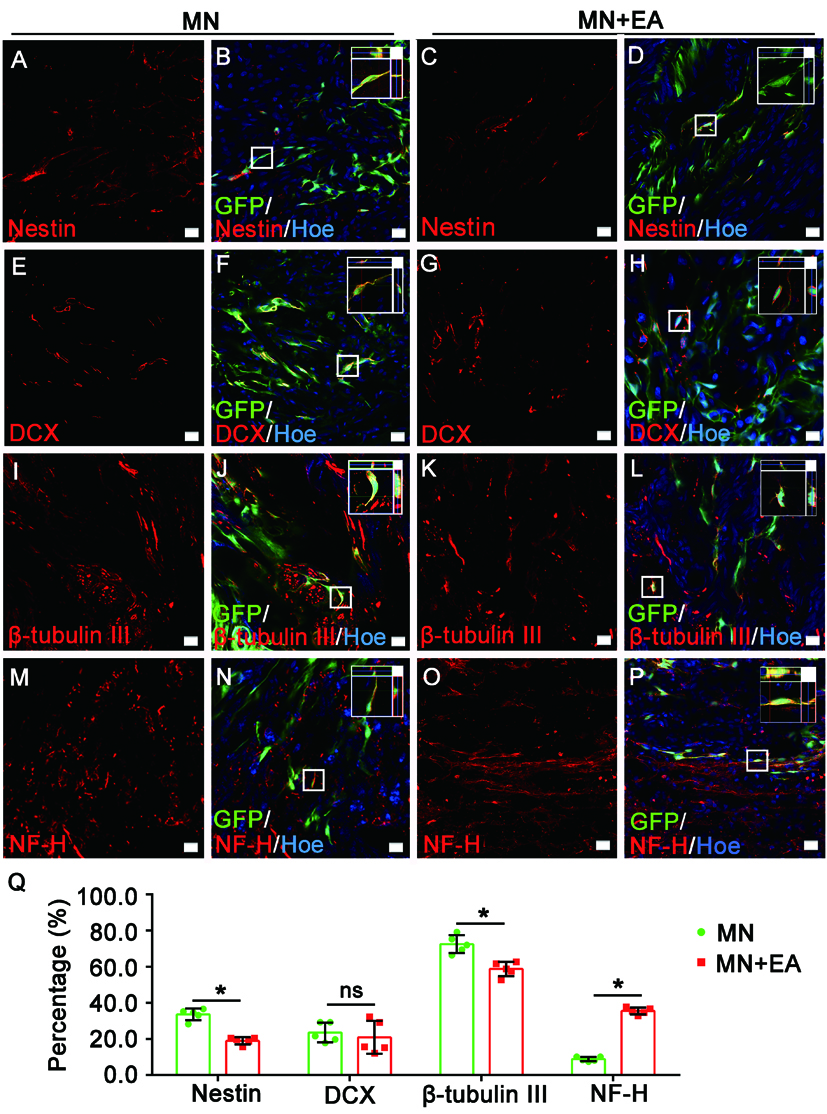


**FIGURE S2** Donor mesenchymal stem cells (MSCs) increased the rate of differentiation into neuron-like cells at the injury/graft site of the spinal cord following electroacupuncture (EA) treatment. (A–P) MSCs differentiated into Nestin-positive neural stem cells (A)–(D), doublecortin (DCX)-positive neural progenitor cells (E)–(H), β-tubulin III-positive immature neuron-like cells (I)–(L), and neurofilament-H (NF-H)-positive neuron-like cells (M)–(P) after 8 weeks of transplantation in the MSC-derived neural network (MN) and MN+EA groups. Inset shows the co-localization between green fluorescent protein (GFP), Hoechst33342 (Hoe), and Nestin (B) and (D), DCX (F) and (H), β-tubulin III (J) and (L) or NF-H (N) and (P). Scale bars = 20 µm in (A)–(P). (Q) Quantification of the percentages of Nestin-, DCX-, β-tubulin III-, and NF-H-positive cells among all MSC-derived cells in the MN and MN+EA groups. Values represent the mean ± SD (*n* = 5/group, Student’s t-test, **p* < 0.05).

**
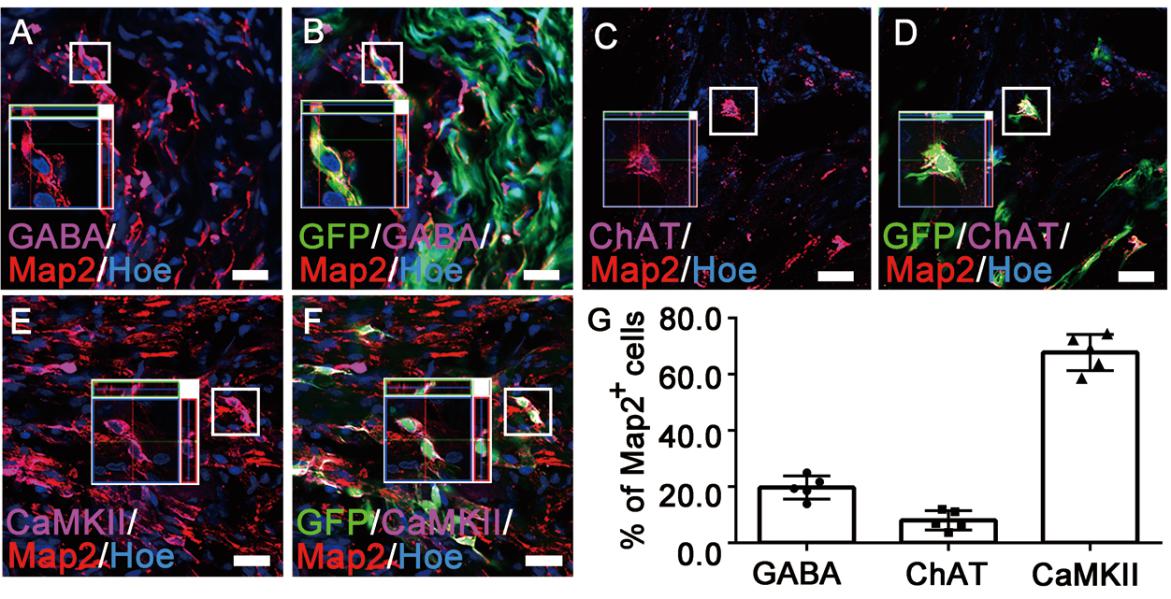
**

**FIGURE S3** Phenotypic characterization of MSC-derived neuron-like cells in the MN+EA group at 8 weeks after transplantation. (A)-(F) Confocal images of neural progenitor cell grafts. Map2 expressing neuron-like cells in grafts express GABA (A) and (B), ChAT (C) and (D), and CaMKII (E) and (F). Insets show co-localization of GFP, Map2 and Hoe with GABA (B), ChAT (D) or CaMKII (F). (G) Bar chart showing the quantification of neuronal subtypes in the MN+EA group. After 8 weeks, most MSC-derived neurons express excitatory neuron markers CaMKII. Values represent mean ± SD. *n* = 5/group. Scale bars = 20 μm in (A)-(F).


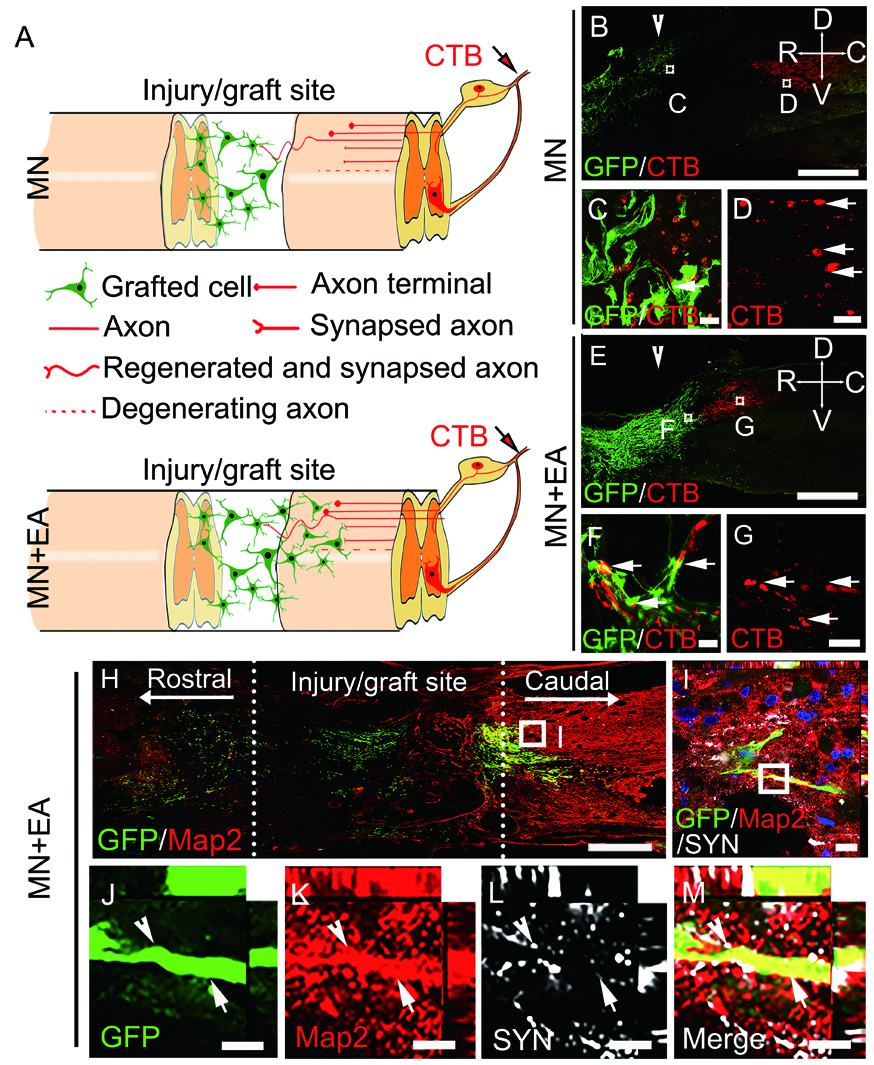


**FIGURE S4** Implanted MSC-derived cells migrated into the caudal spinal cord and made contacts with host caudal neurons. (A) A schematic diagram depicting the labeling of ascending nerve fibers by injecting CTB-555 into the sciatic nerve. (B) and (E) Low-magnification images showing GFP^+^ cells (green) and CTB^+^ ascending nerve fibers (red) in sagittal sections of spinal cord; arrowheads indicate the interface between the injury/graft site and the caudal host tissue, respectively. (C) and (F) Sagittal section of injured spinal cord showing CTB^+^ ascending nerve fibers making potential synaptic contacts (arrows) with GFP^+^ cells. (D) and (G) Sagittal view of injured spinal cord showing CTB^+^ ascending nerve fiber terminals (arrows). (H) A sagittal section showing GFP^+^ MSC-derived neuron-like cells migrated into the host caudal spinal cord tissue and contacted with Map2^+^ nerve fibers (I). (J)-(M) Showing enlarged view of images of boxed area in (I), triple-labeled with anti-GFP (J), Map2 (K) and SYN (L), and their merged image (M). Arrowheads indicate host Map2**^+^**/SYN**^+^** axon terminals contacting with a GFP**^+^**/Map2**^+^** MSC-derived neuron-like cell process. Arrows point at GFP**^+^**/Map2**^+^** MSC-derived neuron-like cell process. D, V, R and C on the right corner in (B) and (E) denote dorsal, ventral, rostral, and caudal to the spinal cord, respectively. Scale bars = 1 mm in (B) and (E); 20 μm in (C)-(D); 500 μm in (H); 10 μm in (I); 5 μm in (J)-(M).


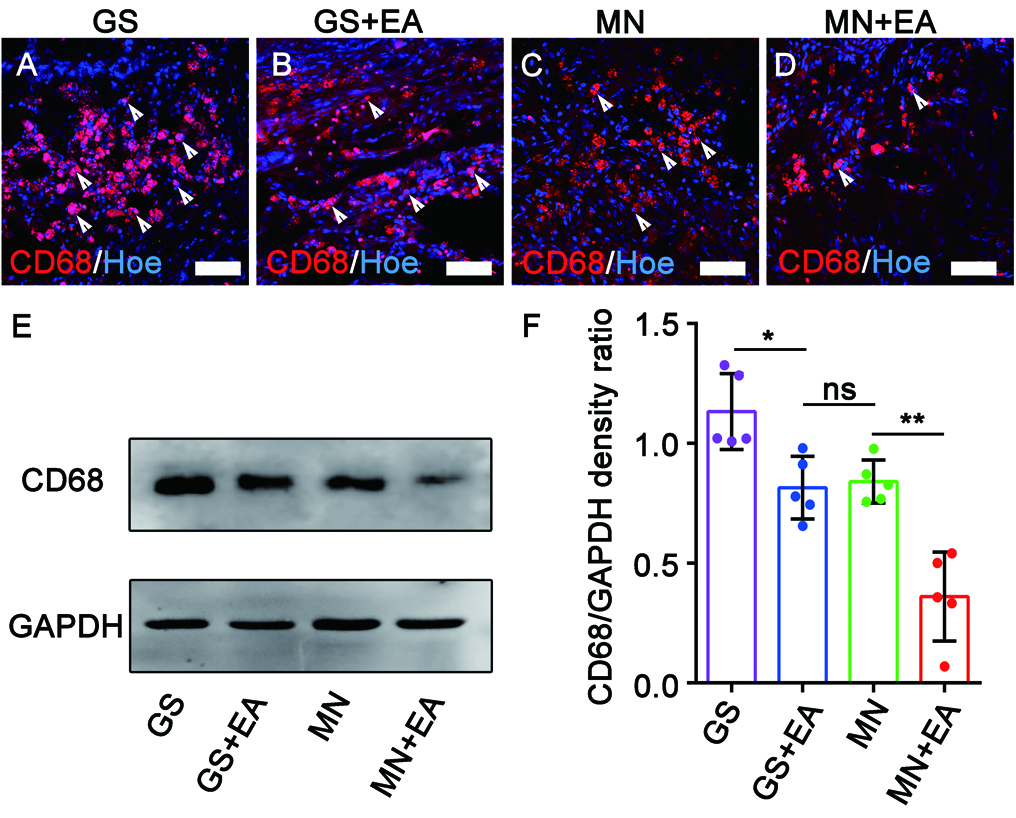


**FIGURE S5** More effective inflammatory alleviation was observed after mesenchymal stem cell (MSC)-derived neural network (MN) implantation combined with electroacupuncture (EA) treatment. (A)–(D) CD68**^+^** cells at the injury/graft site of the spinal cord in the GS, GS+EA, MN, and MN+EA groups. Scale bars = 50 µm in (A)–(D). (E) Western blot analysis showing the protein levels of CD68 in each group. Glyceraldehyde 3-phosphate dehydrogenase (GAPDH) was used as a loading control. (F) Bar chart showing relative quantification of CD68 protein expression in all four groups. Values represent the mean ± SD. *n* =5/group. * and ** indicate *p* < 0.05 and *p* < 0.01, respectively. GS: gelatin sponge scaffold with no cells; GS+EA: GS combined electroacupuncture; MN: MSC-derived neural network; MN+EA: MN combined with electroacupuncture.


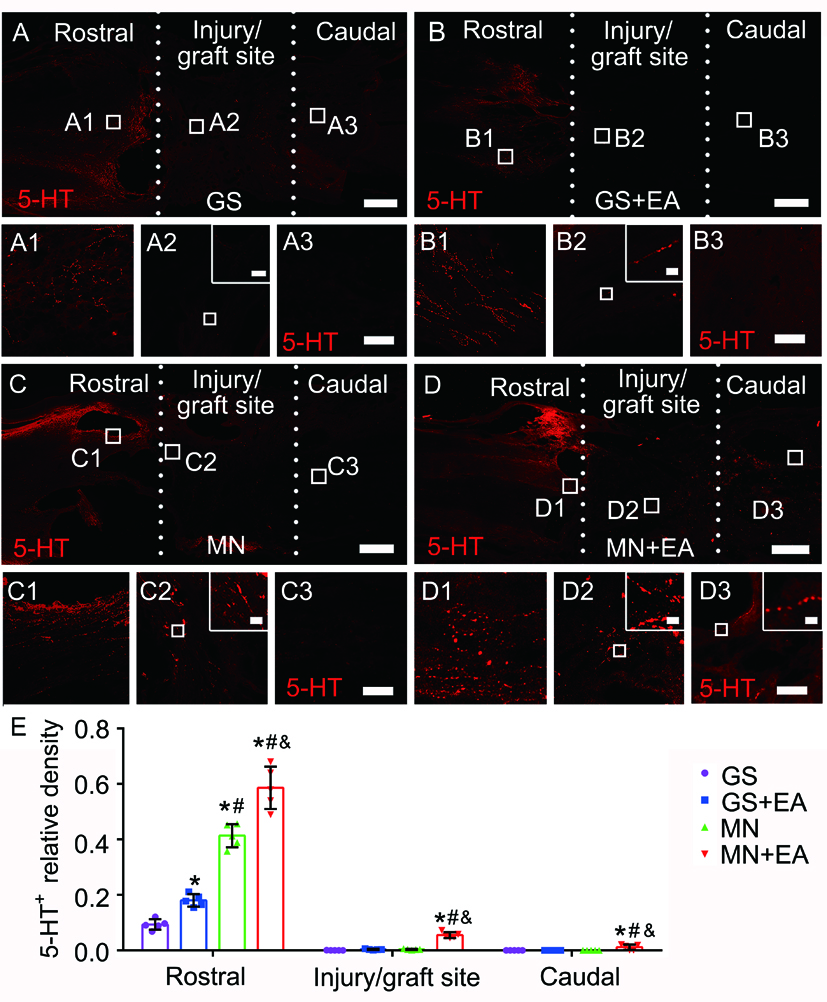


**FIGURE S6** Regeneration of 5-hydroxytryptamine (5-HT)**^+^** axons. (A–D) Longitudinal sections of the spinal cord with 5-HT**^+^** axons in the GS (A), GS+EA (B), MN (C), and MN+EA (D) groups. (A1)–(D3) Immunofluorescent images of the boxes in (A)–(D), respectively. Scale bars = 500 µm in (A)–(D); 50 µm in (A1)–(D3); 10 µm in (F)–(I); 2 µm in (i). (E) Bar chart comparing the quantity of 5-HT**^+^** axons in the rostral and caudal areas relative to the injury/graft site among the four groups. Values represent the mean ± SD (*n* = 5/group, LSD-*t*, **p* < 0.05 *vs.* the GS group, ^#^*p* < 0.05 *vs.* the GS+EA group, ^&^*p* < 0.05 *vs*. the MN group). GS: gelatin sponge scaffold with no cells; GS+EA: GS combined electroacupuncture; MN: MSC-derived neural network; MN+EA: MN combined with electroacupuncture.

**
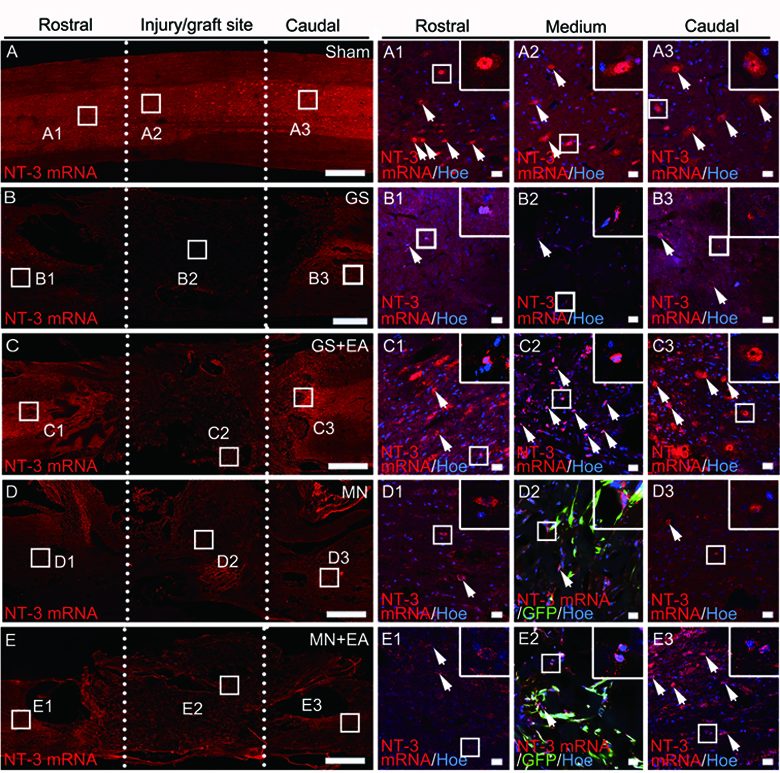
**

**FIGURE S7** *In situ* hybridization analysis showing the distribution of NT-3 mRNA in injured spinal cord. (A)-(E3) Representative images showing the immunostaining of NT-3 mRNA within injured spinal cord in the Sham group (A), (A1)-(A3); GS group (B), (B1)-(B3); GS+EA group (C), (C1)-(C3); MN group (D), (D1)-(D3); and MN+EA group (E), (E1)-(E3), respectively. The cell nuclei were counterstained by Hoechst33342 (Hoe). The insets that showed a magnification (zoom) image of the cells in the tissue have been respectively added in (A)-(E3). Scale bars = 500 μm in (A)-(E); 10 μm in (A1)-(A3), (B1)-(B3), (C1)-(C3), (D1)-(D3) and (E1)-(E3).

**
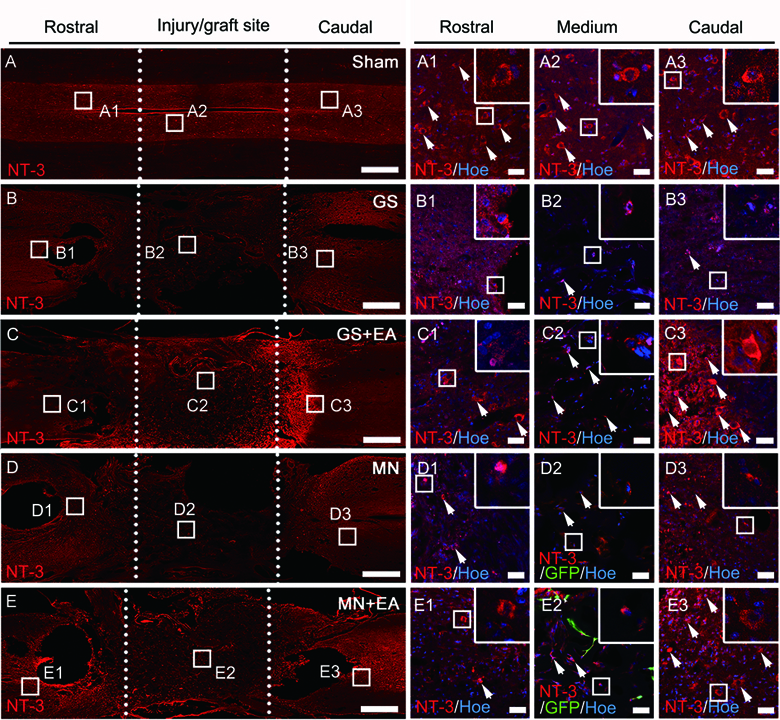
**

**FIGURE S8** Distribution of NT-3 in injured spinal cord. (A)-(E3) Representative images showing NT-3 immunostaining in injured spinal cord in the Sham group (A), (A1)-(A3), the GS group (B), (B1)-(B3), the GS+EA group (C), (C1)-(C3), the MN group (D), (D1)-(D3) and the MN+EA group (E), (E1)-(E3), respectively. The cell nuclei were counterstained by Hoechst33342 (Hoe). The insets that showed a magnification (zoom) image of the cells in the tissue have been respectively added in (A)-(E3). Scale bars = 500 μm in (A)-(E); 20 μm in (A1)-(A3), (B1)-(B3), (C1)-(C3), (D1)-(D3) and (E1)-(E3).

**
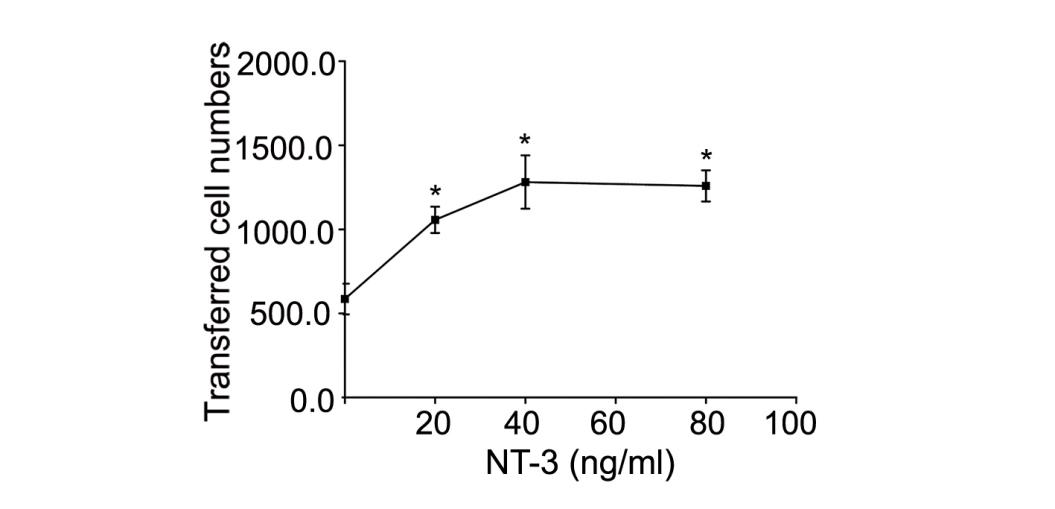
**

**FIGURE S9** Effect of NT-3 on TrkC-MSC migration *in vitro* (48 h). NT-3 attracted TrkC-MSC migration in a dose-dependent manner. Results are presented as mean ± SD. **p* < 0.05 *vs.* Control (NT-3 = 0 ng/ml).

**
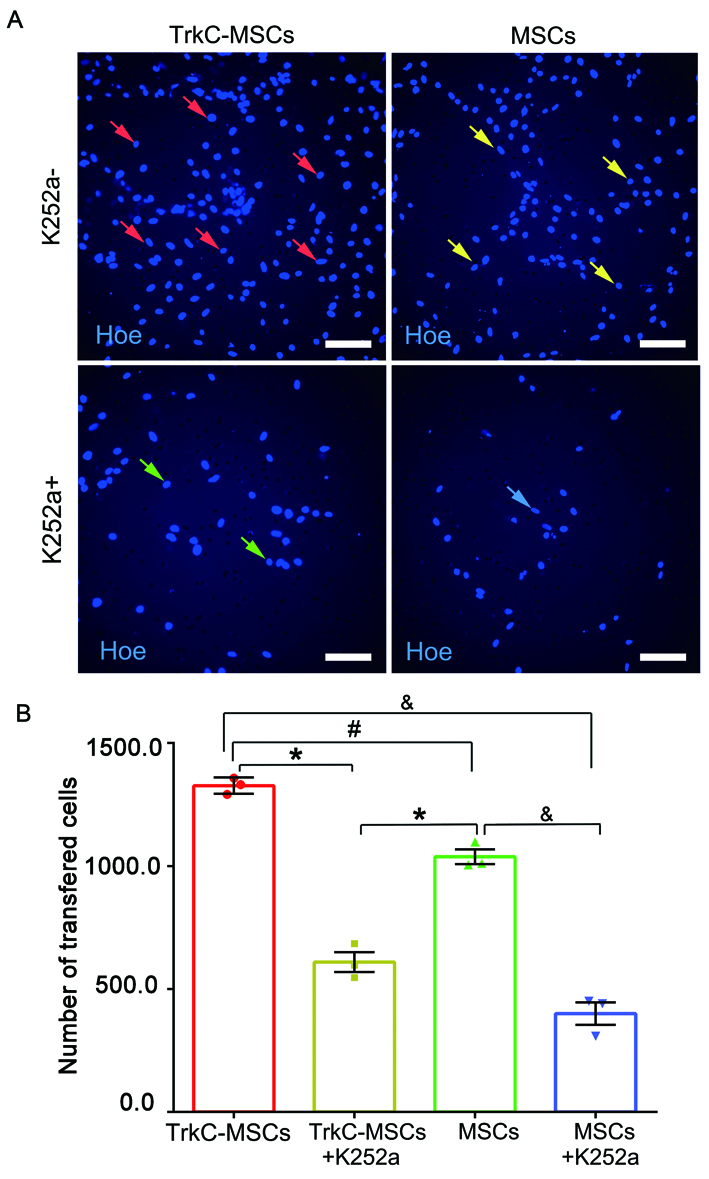
**

**FIGURE S10** Chemotactic migration of TrkC-MSCs. (A) Transwell migration assay showed migration of MSCs and TrkC-MSCs in response to a solution of NT-3 (*c* = 40 ng/ml) with K252a (*c* = 10 nM) or a solution of NT-3 without K252a, respectively. The nuclei of MSCs and TrkC-MSCs were stained with Hoechst33342 (Hoe). Scale bars = 100 μm in (A). (B) Bar chart showing the number of migrating cells in different groups as assessed by Transwell experiment. Results are presented as mean ± S.D., **p* < 0.05 *vs.* TrkC-MSCs+K252a, ^#^*p* < 0.05 *vs.* MSCs, ^&^*p* < 0.05 *vs.* MSCs+K252a.

**Table S1** Primary and secondary antibodies

| Antibodies | Species | Type | Dilution | Source |
| --- | --- | --- | --- | --- |
| Neuroﬁlament 200 (NF) | Rabbit | Polyclonal IgG | 1:400 | Merck Millipore, Billerica, USA |
| Nestin | Mouse | Monoclonal IgG | 1:200 | Abcam, United Kingdom |
| Doublecortin (DCX) | Rabbit | Polyclonal IgG | 1:400 | Abcam, United Kingdom |
| β-tubulin III | Rabbit | Polyclonal IgG | 1:500 | Sigma, St. Louis, USA |
| Neurofilament 200 (NF-H) | Rabbit | Polyclonal IgG | 1:600 | Merck Millipore, Billerica, USA |
| Neurofilament 200 (NF-H) | Mouse | Monoclonal IgG | 1:600 | Sigma, St. Louis, USA |
| CD68 | Mouse | Monoclonal IgG | 1:100 | R&D System |
| Postsynaptic density Protein 95 (PSD95) | Rabbit | Polyclonal IgG | 1:800 | Abcam, London, UK |
| Vesicular glutamate Transporter 2 (VGlut-2) | Rabbit | Polyclonal IgG | 1:1000 | Sigma, St. Louis, USA |
| Microtubule-associated protein 2 (MAP2) | Chicken | Monoclonal IgG | 1:1500 | Abcam, London, UK |
| Green ﬂuorescent protein (GFP) | Rabbit | Polyclonal IgG | 1:500 | Merck Millipore, Billerica, USA |
| Green ﬂuorescent protein (GFP) | Mouse | Monoclonal IgG | 1:1000 | Merck Millipore, Billerica, USA |
| Glyceraldehyde-3-phosphate dehydrogenase(GAPDH) | Mouse | Monoclonal IgG | 1:2500 | Proteintech, Rosemont, USA |
| calcium/calmodulin-dependent protein kinase (CaMKII) | Mouse | Monoclonal IgG | 1:100 | Santa Cruz Biotechnology, USA |
| γ-aminobutyric acid (GABA) | Rabbit | Polyclonal IgG | 1:500 | Boster, Wuhan, China |
| Microtubule-associated protein 2 (MAP2) | Mouse | Monoclonal IgG | 1:1000 | Sigma, St. Louis, USA |
| Microtubule-associated protein 2 (MAP2) | Rabbit | Polyclonal IgG | 1:800 | Sigma, St. Louis, USA |
| Choline acetyltransferase (ChAT) | Rabbit | Polyclonal IgG | 1:800 | Merck Millipore, Billerica, USA |
| Pseudorabies virus (PRV) | Rabbit | Polyclonal IgG | 1:800 | Abcam, London, UK |
| Synaptophysin (SYN) | Mouse | Monoclonal IgG | 1:200 | Sigma, St. Louis, USA |
| Neurotrophin-3 (NT-3) | Rabbit | Polyclonal IgG | 1:300 | Santa Cruz Biotechnology, Santa Cruz, USA |
| 5-hydroxytryptamine (5-HT) | Rabbit | Polyclonal IgG | 1:8000 | Sigma, St. Louis, USA |
| glyceraldehyde-3-phosphate dehydrogenase (GAPDH) | Mouse | Monoclonal IgG | 1:1000 | Proteinteach, Rosemont, USA |
| Horseradish Peroxidase (HRP) | Rabbit | Polyclonal IgG | 1:5000 | Cell Signaling Technology, Boston, USA |
| Horseradish Peroxidase (HRP) | Mouse | Monoclonal IgG | 1:2000 | Cell Signaling Technology, Boston, USA |
| Streptavidin, Alexa Fluor™ 555 conjugate | --- | --- | 1:500 | Invitrogen, Waltham, USA |
| Hoechst33342 (Hoe) | --- | --- | 1:1000 | Beyotime, Shanghai, China |
| Alexa 647 conjuncted anti rabbit secondary antibody | Goat | Polyclonal IgG | 1:500 | Abcam, London, UK |
| Alexa555 conjuncted antirabbit secondary antibody | Goat | Polyclonal IgG | 1:500 | Abcam, London, UK |
| Alexa555 conjuncted antichickensecondary antibody | Goat | Polyclonal IgG | 1:500 | Invitrogen, Waltham, USA |
| Alexa647 conjuncted antichickensecondary antibody | Goat | Polyclonal IgG | 1:500 | Abcam, London, UK |
| Alexa488 conjuncted anti mouse secondary antibody | Goat | Polyclonal IgG | 1:500 | Invitrogen, Waltham, USA |
| Alexa555 conjuncted anti mouse secondary antibody | Goat | Polyclonal IgG | 1:500 | Abcam, London, UK |
